# High performers demonstrate greater neural synchrony than low performers across behavioral domains

**DOI:** 10.1162/imag_a_00128

**Published:** 2024-04-15

**Authors:** Taylor A. Chamberlain, Anna Corriveau, Hayoung Song, Young Hye Kwon, Kwangsun Yoo, Marvin M. Chun, Monica D. Rosenberg

**Affiliations:** Department of Psychology, Columbia University, New York, NY, United States; Department of Psychology, The University of Chicago, Chicago, IL, United States; Department of Psychology, Yale University, New Haven, CT, United States; Department of Neurology, Northwestern University Feinberg School of Medicine, Chicago, IL, United States; Department of Digital Health, Samsung Advanced Institute for Health Sciences and Technology, Sungkyunkwan University, Seoul, South Korea; Data Science Research Institute, Research Institute for Future Medicine, Samsung Medical Center, Seoul, South Korea; Department of Neuroscience, Yale School of Medicine, New Haven, CT, United States; Wu Tsai Institute, Yale University, New Haven, CT, United States; Neuroscience Institute, The University of Chicago, Chicago, IL, United States

**Keywords:** functional MRI, representational similarity analysis, individual differences, intersubject correlation, naturalistic stimuli, attention

## Abstract

Heterogeneity in brain activity can give rise to heterogeneity in behavior, which in turn comprises our distinctive characteristics as individuals. Studying the path from brain to behavior, however, often requires making assumptions about how similarity in behavior scales with similarity in brain activity. Here, we expand upon recent work (Finn et al., 2020) which proposes a theoretical framework for testing the validity of such assumptions. Using intersubject representational similarity analysis in two independent movie-watching functional MRI (fMRI) datasets, we probe how brain-behavior relationships vary as a function of behavioral domain and participant sample. We find evidence that, in some cases, the neural similarity of two individuals is not correlated with behavioral similarity. Rather, individuals with higher behavioral scores are more similar to other high scorers whereas individuals with lower behavioral scores are dissimilar from everyone else. Ultimately, our findings motivate a more extensive investigation of both the structure of brain-behavior relationships and the tacit assumption that people who behave similarly will demonstrate shared patterns of brain activity.

## 1 Introduction

Individuals are amalgamations of their unique traits and abilities, and a key aim of cognitive neuroscience is uncovering how these personal characteristics arise from brain activity. But what is the nature of the relationship between individual differences in behavior and individual differences in brain activity?

If this question seems too theoretical to inform the day-to-day aspects of research, it is worth considering that we often answer it implicitly. Specifically, in modeling task performance or behavioral traits, we frequently assume that people who behave similarly, as measured by similar scores on a given metric, will demonstrate some shared pattern of brain activity. For instance, we might examine brain activity of participants with depression, expecting that depressed participants will exhibit more or less activity (or functional connectivity) in a given region compared to controls (Greicius et al., 2007;Siegle et al., 2002). Another popular method of relating patterns of brain activity to task performance or traits is regression analysis. Regression analyses can identify univariate or multivariate patterns associated with self-report measures, such as the Beck Depression Inventory (BDI) for assessing depression severity (Bezmaternykh et al., 2021;Huang et al., 2021;Trettin et al., 2022;Zhang et al., 2023). In the same vein, if we are interested in individual differences in sustained attention, we may opt to use regression to identify functional connectivity edges that are associated with better or worse attentional performance on a behavioral task (Rosenberg et al., 2016;Yoo et al., 2022).

The idea that people who are similar in some regard will share some aspect of brain function is often assumed. But is it accurate? Returning to the example of depression research, suppose you are collecting fMRI movie watching data, and the patient sample is composed of some individuals with more externalizing symptoms and others with more internalizing symptoms. Perhaps the individuals with greater externalizing symptoms interpret the movie similarly, while those with greater internalizing symptoms are more unpredictable in their narrative interpretations. Consequently, examining a brain activity in a region of interest, there may be greater variability in activity in patients with greater internalizing symptoms. However, correlating activity in this region with the internalizing symptom score might yield a null result, and running a t-test comparing activity in this region for the two subgroups could also yield a null result. In this way, by assuming that individuals with higher internalizing scores will appear similar in terms of brain activity, we run the risk of missing the underlying relationship entirely. (Traditional cognitive tasks are not immune to this potential pitfall; task-data functional connectivity analyses, for instance, could suffer from this same issue.)

Indeed, several studies provide evidence that occasionally behavioral similarity (in the above example, similar clinical profile) accompanies*less*similar brain activity. For instance, work shows that participants with autism spectrum disorder exhibit lower neural synchrony during movie-watching compared to controls (Hasson et al., 2009;Lyons et al., 2020;Salmi et al., 2013), as well as lower intersubject similarity of subnetwork structure (Glerean et al., 2016) and greater variability in intersubject functional correlation states (Bolton et al., 2020). Intersubject correlation during movie-watching is also decreased among individuals with melancholic depression (Guo et al., 2015), attention-deficit/hyperactivity disorder (ADHD) (Salmi et al., 2020), and schizophrenia (Tu et al., 2019) compared to controls. Outside of movie-watching data, work has demonstrated that individuals with autism spectrum disorder show greater inter-individual variability in fMRI activity (as measured by correlational distance between vectorized beta values) during a spatial working memory task (Hawco et al., 2020). Greater variability between individuals may be related to greater variability within individuals over time. Within-subject analyses have revealed that brain-signal variability in the prefrontal cortex scales with ADHD symptom severity in children (Nomi et al., 2018) and that patients with schizophrenia exhibit greater variability in fMRI activity than controls (Gallucci et al., 2022;Maïza et al., 2010;Yang et al., 2014). Individuals with eating disorders also demonstrate increased neuronal variability in the ventral attention network during resting state relative to controls (Spalatro et al., 2019). In sum, a great deal of clinical work has found evidence of increased variability in brain activity between and within individuals diagnosed with autism spectrum disorder, depression, ADHD, schizophrenia, and eating disorders (Dinstein et al., 2015).

Similar behavior, therefore, does not always imply similar brain activity. When should we expect this to be the case? Recent work by Finn and colleagues proposes a paradigm that can help elucidate the complex relationship between brain and behavioral similarity by empirically testing different models of brain-behavior relationships (Finn et al., 2020). The first model, the*Nearest Neighbors*model, operationalizes the assumption that those who score similarly on some behavioral metric (e.g., task performance or questionnaire score) will appear similar in brain activity. Studies testing for a linear brain-behavior relationship assume the*Nearest Neighbors*model. Alternatively, the second model, the*Anna Karenina (AnnaK)*model is named after the opening line of the novel*Anna Karenina*: “Happy families are all alike; every unhappy family is unhappy in its own way.” The*AnnaK*model proposes that one end of a behavioral spectrum is associated with greater variability in brain activity. Finn et al. found that a measure of working memory showed an*AnnaK*-style relationship, where high scorers displayed greater neural synchrony with each other, whereas low-scoring participants were dissimilar to both low and high scorers.

Here, we replicate and extend this finding in two independent fMRI datasets in which youth or adult participants watched movies during scanning. In doing so, we can determine to what extent the best model linking brain and behavioral similarity depends on the characteristics of the sample in question or on the specific behavior or phenotypic measure under investigation. To compute brain similarity, we use intersubject correlation during movie watching, a measure that has been shown to reflect narrative understanding, such that individuals with similar interpretations demonstrate greater intersubject correlation (Nguyen et al., 2019;Yeshurun et al., 2017;Zadbood et al., 2022). In analyzing a developmental dataset, we extend work suggesting that within-individual neural variability changes across development, with older children showing greater variability in EEG signal (McIntosh et al., 2008) and fMRI functional connectivity (Hutchison & Morton, 2015;Marusak et al., 2017) than younger children. Specific to intersubject correlation in development in particular, prior work found that a relationship between depression symptoms and neural synchrony emerges in adolescence, suggesting that brain-behavior relationships may vary as a function of age (Gruskin et al., 2020).

Ultimately, across both the developmental and young adult sample, we find significant evidence for the*AnnaK*model. The*AnnaK*model fits our data across six measures: three in the developmental sample (a working memory task, an attention/executive function task, and a language task) and three in the adult sample (a working memory task, a sustained attention task, and a divided attention/tracking task). In contrast, the*Nearest Neighbors*only explains significant variance in neural similarity for a subset of measures where we find support for the*AnnaK*model. As a whole, our findings illustrate that neural similarity depends not only on whether two individuals resemble each other behaviorally, but also on one’s absolute position on a behavioral scale.

## 2 Methods

### 2.1 Overview

We perform intersubject representational similarity analysis in two datasets with different demographic characteristics (a developmental sample including patients with varied psychiatric diagnoses, and a non-clinical adult sample) and types of movie stimuli (a clip of an animated movie, and an abstract visual-only film). In each of these datasets, we assess the fit of two theoretical models of brain-behavior relationships: the*Nearest Neighbors*model and the*AnnaK*model, to determine which model best describes the data for a given behavioral trait or task. Finally, in examining the results of our two datasets in tandem, we investigate potential similarities in brain-behavior relationships which hold across differences in sample characteristics and stimulus choice.

#### 2.1.1 Dataset 1: Healthy Brain Network

##### 2.1.1.1 Participants

To investigate how similarity in brain activity relates to behavioral similarity in development, we analyzed data from the Healthy Brain Network Biobank (Alexander et al., 2017). This project is collecting data from children and adolescents aged 5-21 years with a diversity of clinical concerns (the majority of the sample having one or more clinical diagnosis). The Healthy Brain Network project was approved by the Chesapeake Institutional Review Board. Secondary data analysis was approved by the University of Chicago Institutional Review Board. Of interest to our current analysis, the dataset includes one fMRI run collected while participants watch an emotionally evocative video, specifically a 10-minute clip from the movie*Despicable Me*. In addition to fMRI data, this sample also contains a large assortment of phenotypic measures, including psychiatric and learning assessments, and questionnaires pertaining to environmental and lifestyle factors. We analyze a subset of these measures in the present study (seeBehavioral Datafor details).

To determine our final study sample, we first downloaded all phenotypic data available as of March 8, 2022 and all available neuroimaging data collected at sites Rutgers and Citibank Biomedical Imaging Center from Healthy Brain Network releases 1-8. Of the participants with downloaded data (*n*= 2131), we subset the sample to those who had complete*Despicable Me*fMRI data and acceptable motion during this scan (defined as maximum head displacement <3 mm and mean framewise displacement <.15 mm) (*n*= 529). Of the participants with a usable*Despicable Me*scan, we retained only participants who had phenotypic data and whose anatomical scan passed visual quality control inspection (*n*= 480).

**Fig. 1.**
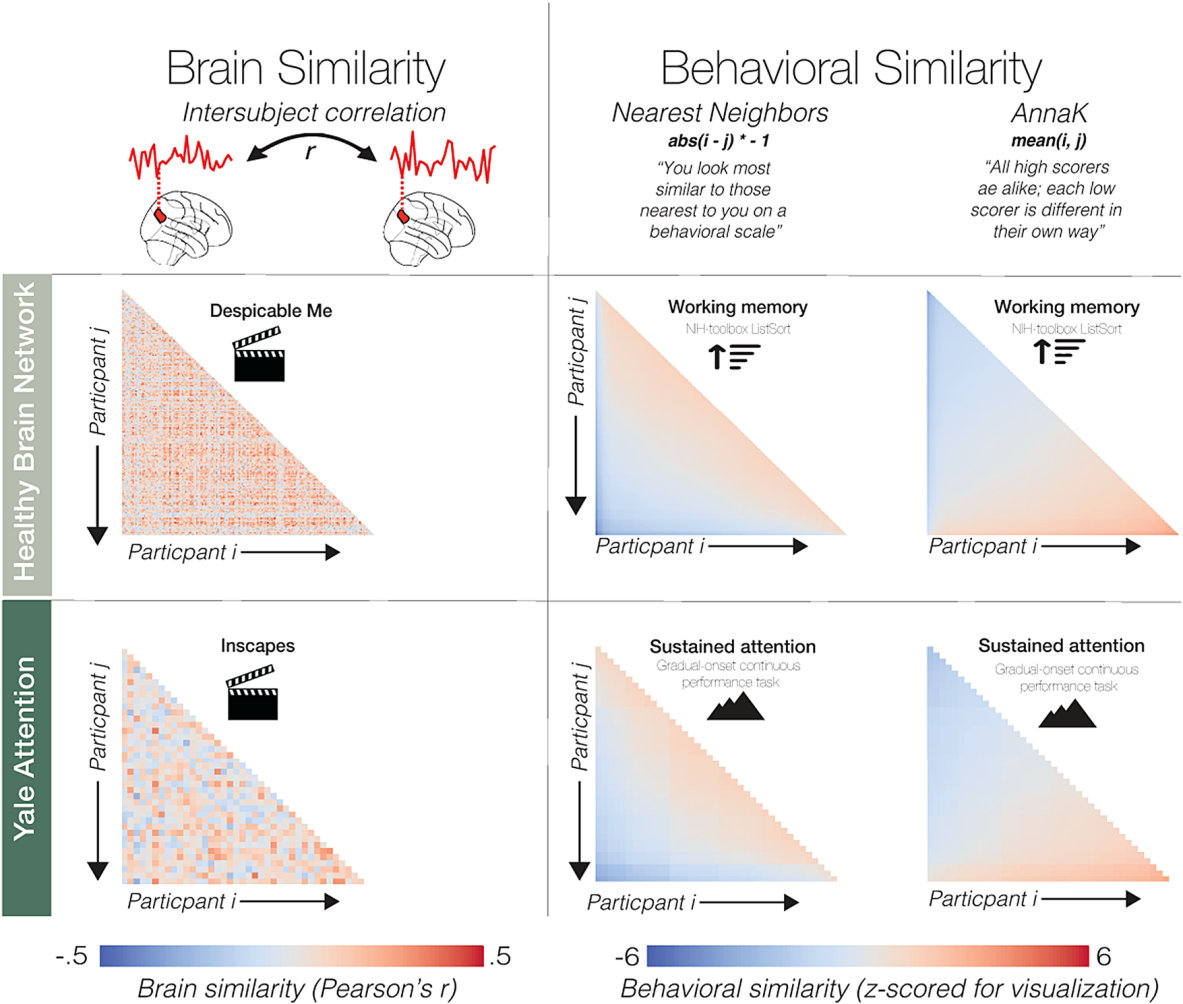
Brain and behavioral similarity matrices. Here, we show similarity matrices, where each row and each column represent a participant. Participants are ordered by behavioral score (ascending). On the left are brain similarity matrices, defined as pairwise intersubject correlation for an example node (node 41) in the Shen parcellation for each dataset. On the right are two different behavioral similarity matrices for the first cohort of each dataset. The first behavioral matrix is calculated according to the*AnnaK*model, while the second is calculated according to the*Nearest Neighbors*model. To assess the relationship between brain similarity and behavioral similarity, we run intersubject representational similarity analysis Spearman correlating the brain and behavioral matrices. Here, we show intersubject RSA results in the three tasks in the Yale Attention dataset. Plotting conventions are the same as inFigure 2.

From this point, phenotypic measures available in 90% of the sample were retained. Of the remaining variables, two raters from our lab selected measures pertaining to five domains of interest selected a priori (cognitive, attention, social, emotional, and language) and grouped these measures by domain. From these measures, a final variable was chosen from each category (see description below). Finally, participants missing any of these measures were excluded, resulting in a final sample of*n*= 409 participants (163 F, 246 M; mean age = 12.26 ± 3.5 years, range = 6–22 years, seeSupplementary Fig. 1for age distribution). The final sample had a mean framewise displacement of .096 mm, with a standard deviation of .026 mm. In the Healthy Brain Network sample, head motion is negatively correlated with age (Pearson correlation*r*(407*)*= -.35,*p *< .001).

##### 2.1.1.2 Behavioral data

**Fig. 2.**
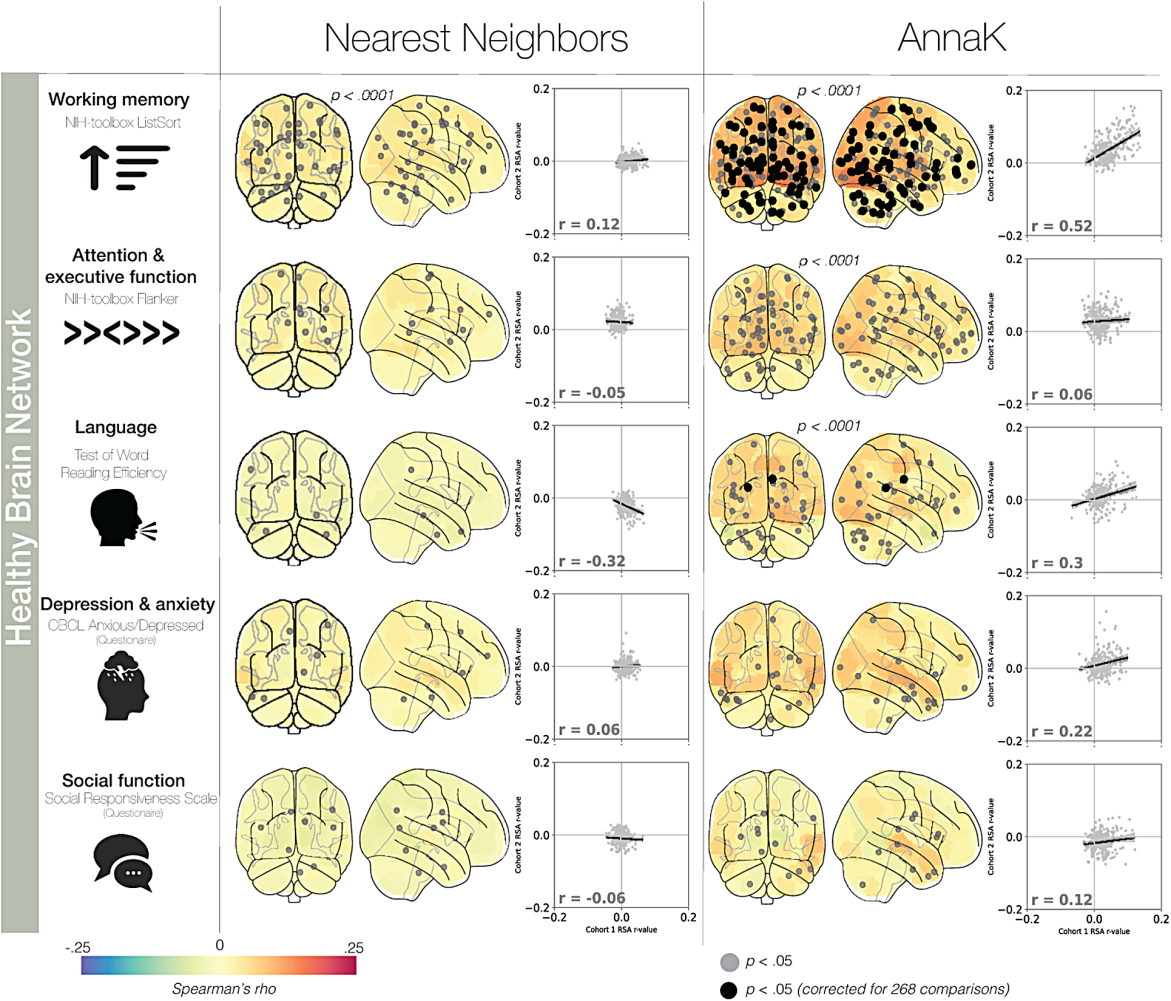
Relating behavior and neural synchrony with intersubject RSA: Healthy Brain Network Sample. In the glass brain, nodes are colored according to representational similarity for each model in our two datasets. Nodes with a black circle at their centroid demonstrate significant representational similarity (*p*< .05, after correction for 268 comparisons). Nodes with a gray circle are significant before multiple comparisons correction (*p*< .05).*p*-Values above the glass brains correspond to the whole-brain familywise*p*-value (seeMethods). Glass brains without*p*-values above all show familywise*p *> .05. The scatterplots to the right of the glass brains show the reliability of the effects as determined by a split-half analysis. The*x*-axis is determined by the representational similarity rho-value in cohort 1 of the split-half analysis. The*y*-axis is determined by the representational similarity rho-value in cohort 2. The regression line and*r*-value labeled in the plot indicate the correlation of the effects across the two cohorts, providing insight into the consistency of the effects across halves of the sample, with a higher correlation indicating greater consistency. Note: the rho-values in the scatterplot are separate from the Spearman’s rho node colors on the glass brain; the former represents effect consistency across all 268 nodes, while the latter denotes the fit of the model in that specific node.

To assess relationships between behavioral similarity and neural synchrony, we took advantage of the large variety of phenotypic data provided by the Healthy Brain Network. The measures analyzed included self-report questionnaires, parent-completed questionnaires, and cognitive assessments such as the NIH toolbox (Hodes et al., 2013). For a complete list of measures analyzed, please seeSupplementary Figure 2.

##### 2.1.1.3 fMRI data acquisition

Data analyzed were collected at one of two sites: Rutgers University Brain Imaging Center or the Citibank Biomedical Imaging Center. Rutgers data were collected on a Siemens 3 T Tim Trio magnet. Citibank Biomedical Imaging Center data were collected on a Siemens 3 T Prisma. Both sites used the following parameters for the functional*Despicable Me*scan: TR = 800 ms, TE = 30 ms, # slices = 60, flip angle = 31, # volumes = 750, voxel size = 2.4 mm^3^, multiband 6. For more information regarding Healthy Brain Network scan parameters, please seeAlexander et al. (2017)andhttp://fcon_1000.projects.nitrc.org/indi/cmi_healthy_brain_network.

##### 2.1.1.4 fMRI preprocessing

AFNI was used to preprocess fMRI data. First, three volumes were removed from each run, followed by despiking and head motion correction. Then, functional images were aligned to the skull-stripped anatomical image with a linear transformation and then to the MNI atlas via nonlinear warping. Covariates of no interest were regressed from the data, including a 24-parameter head motion model (6 motion parameters, 6 temporal derivatives, and their squares) and mean signal from subject-specific eroded white matter and ventricle masks and the whole brain. Finally, images were band-pass filtered from .01 to .1 Hz.

#### 2.1.2 Dataset 2: Yale Attention

##### 2.1.2.1 Participants

To compare our findings in development with that of an independent adult sample, we analyzed a dataset of healthy young adults who participated in a two-session neuroimaging experiment (Rosenberg et al., 2020;Yoo et al., 2022). Sessions were separated by 17.31 days on average (*s.d.*= 20.21 days, median = 12 days;Yoo et al., 2022). This project was approved by the Yale University Human Subjects Committee. Relevant to the present analysis, the participants in this study completed a fMRI run during each session in which they watched the short film*Inscapes*(Vanderwal et al., 2015). This film consists of dynamic visuals with no discernible narrative, making for an interesting comparison to the plot-driven clip shown in the Healthy Brain Network dataset. This film was shown to the participants without sound. Prior to our access of the data, 33 participants were excluded due to unacceptable head motion (>3 mm maximum head displacement and >.15 mm mean framewise displacement), task performances falling 2.5 standard deviations above or below the mean, or because of low-quality imaging data. After accessing the data, an additional 9 scans were dropped due to having greater than 50% of frames censored for motion. This yielded a final sample of*n*= 71 participants (47 F, 24 M; mean age = 22.86 ± 4.28 years, range = 18–36 years). The mean framewise displacement in the final sample is as follows: Session 1 mean: .089, standard deviation: .018; Session 2 mean: .093, standard deviation: .019. This motion is less than that of the Healthy Brain Network sample, although this difference is not significant for the second session (independent-samples t-test Session 1: t_478_= -2.37,*p*= .02, Session 2: t_478_= -1.32,*p*= .19).

##### 2.1.2.2 Behavioral data

Whereas the Healthy Brain Network phenotypic data consist primarily of questionnaires, the Yale Attention dataset includes performance measures from three validated cognitive tasks. In this study, participants completed two sessions of the following three tasks designed to assess working memory and attentional performance: sustained attention measured with the gradual-onset continuous performance task (***gradCPT***), working memory measured with the visual short-term memory task (***VSTM***), and divided attention and tracking measured with the multiple object tracking task (***MOT***). For detailed descriptions of timing and other parameters for all tasks, seeYoo et al. (2022).

The working memory task, the***VSTM***task, is a change detection task measuring visual working memory. During this task, participants viewed an array of 2, 3, 4, 6, or 8 colored circles, which were randomly positioned on the screen. After 100 ms, the circles were replaced by a fixation square for 900 ms before reappearing. In half of the trials, the colors of the circles remained unchanged, and on half the circles reappeared in a different color. Participants had to press one button if they detected a color change and a different button if there had been no change. Performance on this task was measured with Pashler’s K (Pashler, 1988).

The sustained attention task, the***gradCPT***(Esterman et al., 2013) assesses participants’ sustained attention and inhibitory control function. In this task, city and mountain photographs are displayed and participants were instructed to press a button in response to city scenes (appearing on 90% of trials) and withhold responses when mountain scenes appear (10% of trials). Images gradually transition from one to the next at a rate of 800 ms/trial. Performance was assessed by mean sensitivity (*d’*).

Finally, the divided attention and tracking task, the***MOT***task*,*assesses attentional selection and tracking (Luck & Vogel, 1997) and was adapted from code fromLiverence and Scholl (2012). On each trial, 12 white circles appeared. Three or five of the circles (the targets) blinked green briefly before all circles began to move around the screen. Participants had to track the locations of the targets until the display stopped moving. When it stopped, one circle flashed, and participants pressed a button to indicate if that circle had been a target. Performance was assessed by mean accuracy across all trials.

##### 2.1.2.3 fMRI data acquisition

Data were collected at the Yale Magnetic Resonance Research Center and Brain Imaging Center on a 3 T Siemens Prisma with a 64-channel head coil. The following parameters were used for the functional scan analyzed here (*Inscapes*movie-watching): TR = 1,000 ms, TE = 30 ms, # slices = 52, flip angle = 62°, # volumes = 600, voxel size = 2.5 mm^3^, and multiband 4. For more information regarding the scan parameters of this data set, please seeYoo et al. (2022).

##### 2.1.2.4 fMRI preprocessing

AFNI was used to preprocess fMRI data. First, three volumes were removed from each run. Then, censoring was performed, removing volumes in which greater than 10% of voxels were outliers, or for which the Euclidean norm of the head motion parameter derivatives was greater than .2 mm. Despiking, slice-time correction, motion correction, and regression of mean signal from the CSF, white matter, and whole brain was performed. Additionally, 24 motion parameters (6 motion parameters, 6 temporal derivatives, and their squares) were regressed from the data. Functional images were aligned to the skull-stripped anatomical image with a linear transformation, and to the MNI atlas via nonlinear warping.

### 2.2 Pairwise intersubject time-course correlation

For every individual in each dataset, preprocessed BOLD signal time-courses were averaged across voxels within regions of interest using a 268-node whole-brain parcellation (Shen et al., 2013). For every node, each participant’s BOLD signal time-course was*z*-scored within-subject and Pearson correlated with that of every other individual in the cohort. This yields a participant-by-participant “brain-similarity” matrix for each node, with each cell representing the synchronization of activation in that node for two individuals. We repeated this process in the Healthy Brain Network sample and the Yale Attention sample.

### 2.3 Intersubject representational similarity analysis

After creating intersubject correlation matrices for each node and dataset cohort, we implemented intersubject representational similarity analysis (IS-RSA) to test two models of brain-behavior relationships: 1) a*Nearest Neighbors*model hypothesizing that individuals with similar behavioral PC scores will show greater brain similarity and 2) an*Anna Karenina (AnnaK)*model stating that individuals with high behavioral scores will show greater brain similarity whereas low scorers will show lower brain similarity (or vice versa) (Chen et al., 2020;Finn et al., 2020;van Baar et al., 2019). The goal of this approach is to determine which of the models, if either, explains the observed relationship between brain similarity and behavioral similarity.

To do this, we computed three participant-by-participant matrices: one for brain similarity (pairwise intersubject correlation), and two for behavioral similarity (seeFig. 1). For the*Nearest Neighbors*model*,*behavioral similarity is defined as the absolute value of the difference in behavioral scores, multiplied by negative one (meaning larger values indicate greater similarity). For the*AnnaK*model, behavioral similarity is the mean of the behavioral scores (note that other operationalizations of this model exist, seeFinn et al., 2020).

**Fig. 3.**
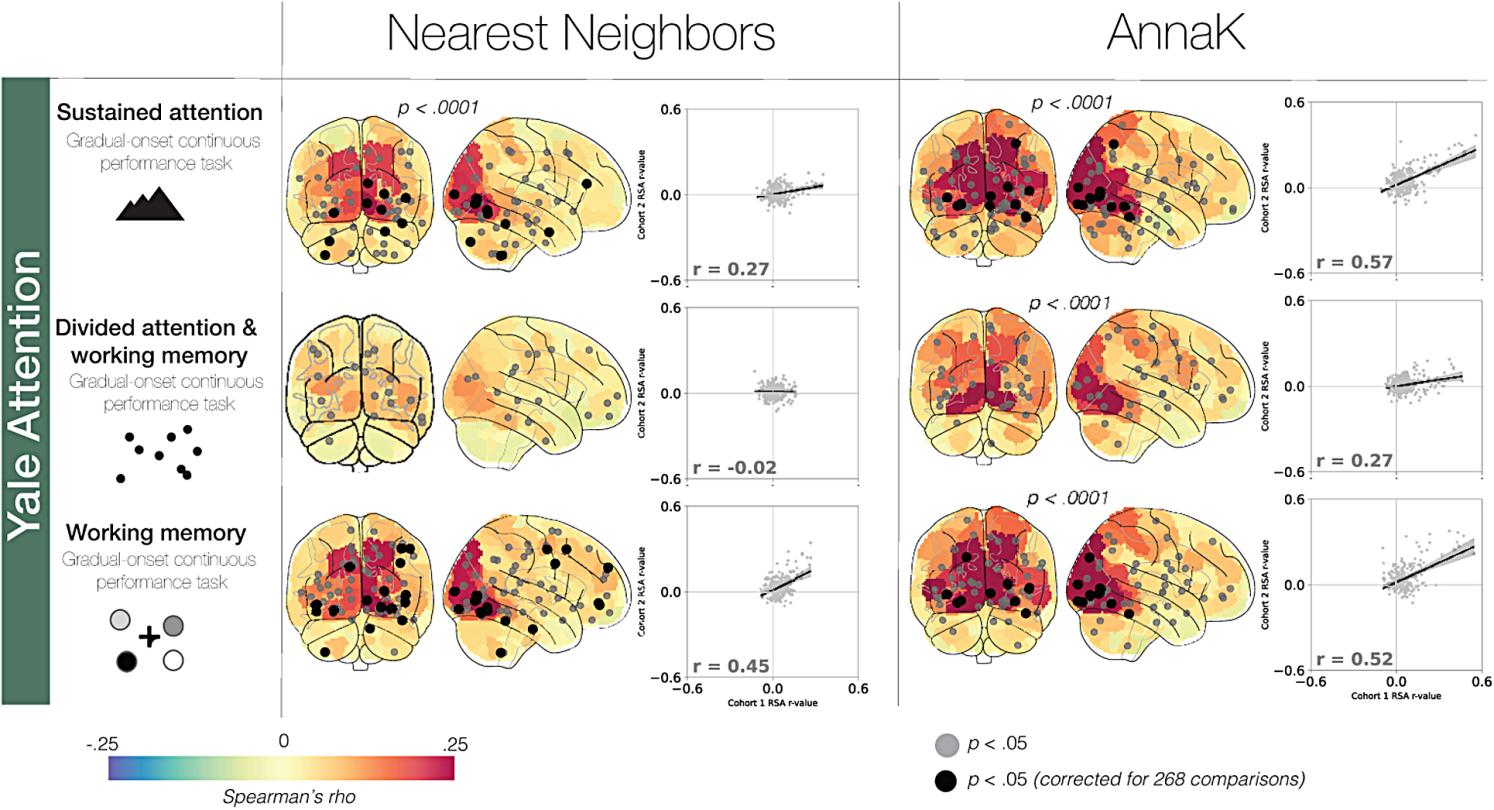
Relating behavior and neural synchrony with intersubject RSA: Yale Attention Sample.

To test how well our models capture brain-behavior relationships, we applied Spearman partial correlation to relate the vectorized lower triangles of the symmetric brain and behavioral similarity matrices, controlling for participant age and sex. To control for participant age and sex, we created three additional matrices: 1) a sex matrix (where a cell contains a one if participants*i*and*j*have the same sex, zero otherwise), 2) an*AnnaK*age matrix (where a cell contains the average of participant*i*and participant*j*’s ages), 3) a*Nearest Neighbors*age matrix (where a cell contains the absolute value of the difference of participant*i*and participant*j*’s ages), 4) an*AnnaK*motion matrix (where a cell contains the average of participant*i*and participant*j*’s mean frame-wise displacement), and 5) a*Nearest Neighbors*motion matrix (where a cell contains the absolute value of the difference of participant*i*and participant*j*’s mean frame-wise displacement). These control matrices and interaction of the age and sex matrices were regressed out of the brain and behavioral similarity matrices, and the subsequent analysis was performed on the residuals (to see a version of the analysis without control matrices regressed out, seeSupplementary Fig. 3). Ultimately, the primary measure of interest is the Spearman correlation between the residualized brain and behavioral similarity matrices (calculated using a Python implementation of the Mantel testhttps://github.com/jwcarr/mantel). This correlation indicates how well the*AnnaK*and*Nearest Neighbors*models, respectively, fit the observed brain data. This correlation is performed in each of the 268 nodes in our parcellation for the IS-RSA of intersubject correlation. It is worth noting that both a positive and negative Spearman’s rho is an interpretable effect for the AnnaK model. A positive correlation would indicate that greater behavioral values are associated with higher neural synchrony. A negative correlation signifies that lower behavioral values are correlated with higher neural synchrony.

To determine significance of model fit for each node, we performed permutation testing (10,000 permutations). For each permutation, the subject labels of the behavioral matrix were randomly shuffled, and the correlation between brain and behavioral matrices was re-calculated, generating a null distribution of 10,000 Spearman rho-values for each node. After using the null distribution to calculate a two-tailed*p*-value for every node, the Holm-Bonferroni method was used to correct for multiple comparisons. To test whether there is greater evidence across the whole brain than would be expected by chance, we used an approach akin to the familywise error control method described inFinn et al. (2020). We randomly generated*p*-values for each of our 268 nodes, calculated the number of nodes which survive a*p*-threshold of .05, and repeated this 10,000 forming a null distribution. We compare the observed number of significant nodes to this null distribution to obtain a whole-brain*p*-value. Note, that whileFinn et al. (2020)performed this calculation for a split-half analysis, in our calculation we use the whole sample.

In order to assess the consistency of each model’s effects, we repeated the calculation of Spearman’s rho for each model in a split-half fashion. Participants were randomly divided into two cohorts, and intersubject RSA was performed separately on each cohort. This analysis allows us to determine how similar the rho-values for a given node are across the two cohorts.

Finally, we directly compared*AnnaK*and*Nearest Neighbors*, testing if we find greater evidence for either model when averaging effects across the whole brain. To do so, we first took the difference in the Fisher-Z transformed rho-values for each node (subtracting the*Nearest Neighbors*Fisher-Z value from the*AnnaK*Fisher-Z value). Next, we averaged this difference across all 268 nodes to get a single difference value, at which point we applied the inverse Fisher-Z transform. To assess significance, we repeated this process 10,000 times, each time shuffling the subject labels of the behavioral matrix, to create a null distribution. Using this null distribution, we calculate a two-sided, non-parametric*p*-value for each behavioral measure in each of our two datasets.

## 3 Results

### 3.1 Principal component analysis of behavioral measures in *Healthy Brain Network* data

**Fig. 4.**
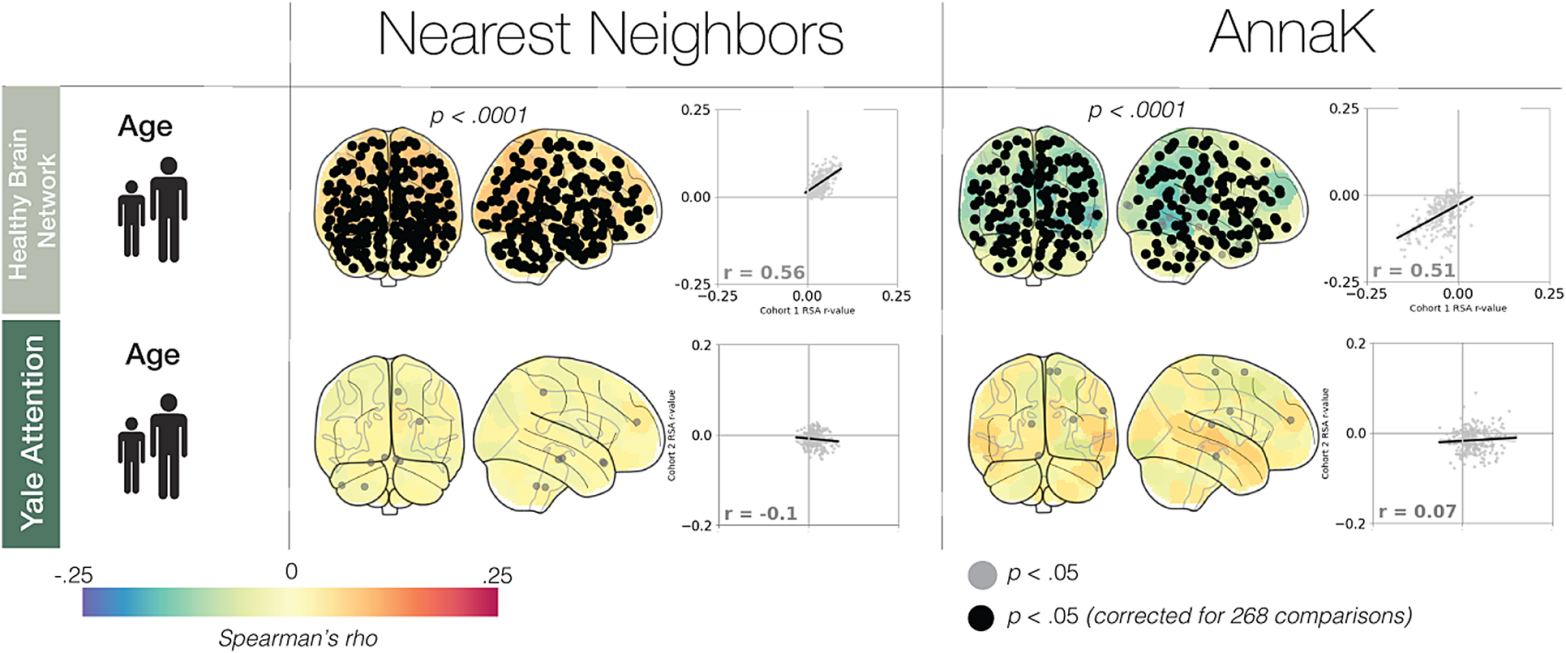
Relating age and neural synchrony with intersubject RSA. In the glass brain, nodes are colored according to representational similarity for each model in each dataset. Nodes with a black circle at their centroid demonstrate significant representational similarity (*p*< .05, after correction for 268 comparisons). Nodes with a gray circle are significant before multiple comparisons correction (*p*< .05).*p*-Values above the glass brains correspond to the whole-brain familywise*p*-value (seeMethods). Glass brains without*p*-values above all show familywise*p *> .05. The scatterplots to the right of the glass brains show the reliability of the effects as determined by a split-half analysis.

After dividing the behavioral measures into five broad behavioral domains (seeSupplementary Fig. 2), we applied principal component analysis (PCA) to the measures in each behavioral domain in the Healthy Brain Network sample. We examined the resulting components, finding that the first principal component explained 47%, 39%, 40%, 68%, and 47% of variance, for the cognitive, social, emotional, attentional, and language domains, respectively (Supplementary Fig. 2). From here, we retained the measure from each domain that explained the most variance in the first components, with two exceptions. For the attention domain, we chose to use the Flanker Inhibitory Control and Attention Test, as it provides a behavioral task measure of attention function, rather than a questionnaire measure of attention, complementing the task measures of attention from the Yale Attention sample. For the cognitive domain, we chose to retain the NIH List Sorting Working Memory Test measure to directly replicate past work in adults using the same measure (Finn et al., 2020) (alternatively, to see results for each “cognitive” variable seeSupplementary Fig. 4). For an alternative analysis using the first principal component as a summary score, seeSupplementary Figure 5.

Questionnaire measures were retained for two domains, social (the Social Responsiveness Scale, measuring social function) and emotional (Child Behavior Checklist Anxious/Depressed subscale, measuring anxiety and depression symptoms). We reverse-coded these measures so that larger values indicate less severe social impairment and anxiety and depressive symptoms, respectively. Task performance measures were retained for three domains, cognitive (NIH Toolbox List Sorting Working Memory Test, measuring verbal working memory), attentional (NIH Toolbox Flanker Inhibitory Control and Attention Test, measuring attention and executive function), and language (Test of Word Reading Efficiency [TOWRE], measuring reading accuracy and fluency). Higher task performance scores indicate better performance. To see correlations between task, age, and head motion, seeSupplementary Figure 6. Throughout the results, we refer to these five retained measures as working memory, attention/executive function, language, depression/anxiety, and social function.

We did not apply PCA to the behavioral measures from the Yale Attention dataset as there were only three measures in total, which assessed aspects of attention and working memory.

### 3.2 Pairwise intersubject time-course correlation

To ask if we can better understand individual differences in behavior by examining intersubject correlation (ISC), we first need to establish that the fMRI BOLD activity time-courses of the movie watching scans are correlated, as we would anticipate. To perform this preliminary check, we averaged ISC values across all pairs of participants for each node in our 268 node parcellation (Supplementary Fig. 7). Average ISC across the whole brain was positive in both datasets and no nodes exhibited negative average ISC (Healthy Brain Network: mean*r*= .062, range:*r*= [.008, .249]; Yale Attention: mean*r*= .038, range:*r*= [.014, .268]). Overall, we observed the highest levels of ISC in regions predicted to be the most synchronized during movie watching: areas associated with visual and auditory processing. For instance, in the Healthy Brain Network sample, which included an audiovisual movie stimulus, the nodes with the five highest ISC values were in one of three areas: extrastriate cortex (*r*= .11, .13), Wernicke’s area (*r*= .10, .18), and visual association cortex (*r*= .12). In the Yale Attention sample, which included a visual-only movie, the five nodes with the highest ISC were all within the primary visual cortex (*r*= .19, .20, .22, .25, .27).

### 3.3 Intersubject representational similarity analysis

#### 3.3.1 Intersubject RSA reveals significant representational similarity across behavioral domains in the Healthy Brain Network sample

Previous work analyzing a working memory task in young adults demonstrated greater evidence for an*AnnaK*model than the*Nearest Neighbors*model (Finn et al., 2020). In other words, participants who scored similarly did not always display greater neural synchrony during movie watching. Rather, high scorers on the working memory task appeared more synchronized with high scorers and while low scorers were less in-sync with all others. Here, we first asked whether this result is idiosyncratic to either the particular sample studied or to the particular type of movie analyzed. For instance, is the*AnnaK*effect dependent on the video shown containing a narrative arc? One possibility is that participants who score highly on a behavioral measure may interpret the narrative similarly in a way that is reflected in their BOLD activity*.*If this is the case, we would not expect to find evidence for the*AnnaK*model in the Yale Attention dataset, which uses*Inscapes*, a film with no narrative or plot to interpret. We then asked which model best explains the relationship between brain similarity and similarity in different types of behaviors. One possibility is that the*AnnaK*model will generally outperform the*Nearest Neighbors*model across a variety of behavioral domains. Alternatively, it is possible this effect is specific to working memory function, the measure assessed in previous work using data from the Human Connectome Project (Finn et al., 2020). If the*AnnaK*model is primarily a good fit for working memory measures, we would expect to find the*AnnaK*model to be a good fit for only two out of the eight behavioral measures analyzed presently: the Healthy Brain Network working memory task (NIH toolbox ListSort) and Yale Attention working memory task (VSTM). Furthermore, since prior work used an adult sample, it is also possible that the*AnnaK*model does not describe brain–behavior relationships in development. In this case, we would not expect to find an*AnnaK*effect in the Healthy Brain Network working memory task.

To investigate, we analyzed brain-behavior relationships in eight behavioral measures using two separate movies viewed in scanner and two independent samples (seeFig. 2). In the Healthy Brain Network sample, the*AnnaK*model captures brain-behavior relationships in more nodes than would be expected by chance for the working memory task, attention/executive function task, and the language task (familywise*p < .*0001). Of the nodes demonstrating an*AnnaK*effect, 76 nodes in the working memory task, and two nodes in the language task nodes survive Bonferroni correction for 268 comparisons. Conversely, the*Nearest Neighbors*model only captures brain-behavior relationships in more nodes than expected by chance for the working memory task (familywise <.001, all other familywise*p *> .95). Additionally, no nodes show a significant*Nearest Neighbors*relationship after multiple comparisons correction. For all five behavioral measures, we observe more significant nodes (uncorrected*p *< .05) under the*AnnaK*model than the*Nearest Neighbors*model (difference in # of significant nodes,*AnnaK*–*Nearest Neighbors:*working memory: 100, attention/executive function: 49, language: 30, depression/anxiety: 11, social function: 3). Finally, examining the split-half consistency of the effects for each model, the*AnnaK*model exhibits greater replicability than the*Nearest Neighbors*model for all five domains (*AnnaK*r_cohort 1, cohort 2_working memory: .52, attention/executive function: .06, language: .3, depression/anxiety: .22, social function: .12,*Nearest Neighbors*r_cohort 1, cohort 2_: working memory: .12, attention/executive function: -.05, language: -.32, depression/anxiety: .06, social function: -.06).

The*AnnaK*model captures brain-behavior relationships in more nodes than would be expected by chance for all three task measures (familywise*p *< .0001), but not the questionnaire-based measures of social function and depression/anxiety symptoms. More research is needed to determine if this difference in results is due to the nature of the measurement (task vs. questionnaire) or the nature of the underlying behavioral or mental processes. Preliminary evidence points to the former hypothesis, as prior research examining a non-self-report social measure, number of social connections reported by one’s peers, found a robust*AnnaK*-effect in several brain regions (Baek et al., 2022).

Of all behavioral measures and models tested, the*AnnaK*model significantly reflects brain-behavior relationships for the most nodes in the working memory task, followed by the attention/executive function task. As an exploratory analysis testing the extent to which the*AnnaK*model fits other cognition-related measures, we took each variable comprising the cognitive PCA (seeMethods) and replicated the IS-RSA with these variables individually (Supplementary Fig. 4). Out of the 12 variables tested, eleven demonstrate a better fit with the*AnnaK*model judging by the number of significant nodes. Consequently, the*AnnaK*effect in the cognitive domain does not appear to be specific to the cognitive tasks shown here.

As a final method of assessing the models, we computed a whole-brain summary score by averaging all nodes’ rho-values. Judging by the difference in this summary score, the*AnnaK*model numerically outperformed the*Nearest Neighbors*model in every behavioral measure tested in the Healthy Brain Network sample. Comparing this difference score to a permuted null distribution of difference scores, the*AnnaK*model better captured the relationship between brain and behavioral similarity than the*Nearest Neighbors*model in for the working memory and attention/executive function tasks (*p*= .027 and*p*= .017).

In sum, across all five behavioral domains in the Healthy Brain Network sample, we found more support for the*AnnaK*model than the*Nearest Neighbors*model, and we found significant evidence for the AnnaK model across all task measures (working memory, attention/executive function, and language). We found support for*Nearest Neighbors*-style relationships in only one behavioral measure: working memory. The positive rho-values of the*AnnaK*effect suggest that participants who score higher on assessments of working memory, attention, and reading ability are more synchronized during movie-watching while those who score lower are less synchronized.

#### 3.3.2 Intersubject RSA reveals significant representational similarity across behavioral domains in the Yale Attention sample

Turning to the results in the Yale Attention Dataset, the*AnnaK*model reflected brain-behavior relationships in all three tasks, as evidenced by more significant nodes than would be expected due to chance (familywise*p < .*0001, seeFig. 3). The*Nearest Neighbors*model significantly reflected brain-behavior relationships for sustained attention (gradCPT) and working memory (VSTM) (familywise*p < .*0001), but not MOT (familywise*p*= .13), performance. For two out of three tasks, we observed more significant nodes (uncorrected*p *< .05) under the*AnnaK*model than the*Nearest Neighbors*model (difference in # of significant nodes by domain,*AnnaK*-*Nearest Neighbors:*sustained attention [gradCPT]: 4, divided attention/tracking [MOT]: 3, working memory [VSTM]: -2). Of the two domains with nodes surviving multiple comparisons correction, both showed more significant nodes under the*AnnaK*model (difference in # of significant nodes by domain,*AnnaK*-*Nearest Neighbors:*sustained attention [gradCPT]: 4, working memory [VSTM]: 3). Finally, the effects of the*AnnaK*model demonstrated greater split-half consistency for all three behavioral tasks (difference in consistency by domain,*AnnaK*r_cohort 1, cohort 2_-*Nearest Neighbors*r_cohort 1, cohort 2_: sustained attention [gradCPT]: .30, divided attention/tracking [MOT]: .29, working memory [VSTM]: .07).

Examining the whole-brain summary analysis in the Yale Attention sample, the AnnaK model significantly outperformed the Nearest Neighbors model in the sustained attention measure (*p*= .025), with stronger numerical effects in the other two measures, divided attention/tracking (*p*= .078) and working memory (*p=*.079).

Ultimately, relative to the*AnnaK*model, we found less compelling evidence for the*Nearest Neighbors*model, a model grounded in the intuitive hypothesis that people who are more similar on a behavioral scale will show more similar brain responses. Rather, on the whole, we found more evidence that brain-phenotype relationships examined showed an*AnnaK*-style pattern, wherein high scorers were synchronized with other scorers while low-scoring participants showed less synchrony across the board. Although more research is necessary to determine under what conditions the*AnnaK*model describes brain-behavior relationships, here we demonstrate that this effect is robust to sample demographics (e.g., children and adolescents vs. adults), movie type (e.g., narrative vs. non-narrative), and the behavioral measure itself (e.g., working memory vs. sustained attention).

#### 3.3.4 Anatomical distribution of intersubject RSA effects differs by dataset

Aside from how well the*AnnaK*and*Nearest Neighbors*models describe the relationships between brain and behavior, we were also curious as to*where*in the brain these models fit the data well. One possibility is that we find more significant nodes in areas where ISC is high; this would mean auditory processing areas for the Healthy Brain Network dataset and visual processing areas for both datasets (the Yale Attention stimuli did not have audio). Alternatively, if ISC is*too*high, it may not provide enough variance to successfully relate to individual differences in behavior. The nature of the stimulus type may also modulate where each model of representational similarity fits. For instance, synchrony in higher-order regions may prove more important when the video stimulus contains more emotional or narrative content, such as the clip shown to the Healthy Brain Network participants. Finally, the distribution of nodes could depend on the model in question, with some regions exhibiting more*AnnaK*or*Nearest Neighbors*-style relationships respectively.

To answer this question, we displayed nodes demonstrating significant representational similarity (uncorrected*p*< .05) within a given model according to which network they belong to (Supplementary Fig. 8). Looking at the*Nearest Neighbors*model in the Healthy Brain Network sample, we observed more nodes than would be expected by chance in the motor network. For the*AnnaK*model, we observed more nodes than would be expected by chance in the Visual I network. In the Yale Attention sample, every behavior for both models showed the greatest number of nodes in the visual cortex. There were more significant nodes in the primary visual cortex than would be expected by chance for both the sustained attention (gradCPT) and working memory (VSTM) tasks across both models (all*p*< .001) and for the divided attention/tracking task (MOT) in the*AnnaK*model (*p*< .01). The Yale Attention sample has a high concentration of nodes in early visual areas across all measures and both models, while the Healthy Brain Network exhibits effects less consistently concentrated in the primary visual cortex.

#### 3.3.5 Model fit is correlated with intersubject correlation

To better understand how our power for detecting individual differences varies as a function of ISC, we also assessed the extent to which the intersubject RSA model fit is correlated with average ISC (Supplementary Figs. 9, 10). If neural synchrony is associated with greater power for picking up on individual differences, we would expect a positive correlation between ISC and model fits. Indeed, this is what we observed. In every instance where the intersubject RSA was significant at the whole-brain level, we see a strong positive correlation between node-wise ISC and node-wise IS-RSA rho (**Health Brain Network***AnnaK*: working memory:*r*= .86 attention/executive function:*r*= .63, language: .53;*Nearest Neighbors*: working memory:*r*= .57;**Yale Attention***AnnaK:*sustained attention [gradCPT]:*r*= .87, divided attention/tracking [MOT]:*r*= .77, working memory [VSTM]:*r*= .87;*Nearest Neighbors*: sustained attention [gradCPT]:*r*= .77, working memory [VSTM]:*r*= .80). In fact, in 14 out of 16 instances (2 models x 8 behavioral measures), we observed a positive correlation between ISC and IS-RSA effects. Here, perhaps counterintuitively, greater similarity across subjects’ brain activity appears to better allow us to understand behavioral differences, consistent with prior work showing that movie data are better than rest data for predictive modeling of individual differences in behavior (Finn & Bandettini, 2021).

#### 3.3.6 Both AnnaK and Nearest Neighbors models significantly describe the relationship between neural similarity and age in development

The*AnnaK*model significantly captured brain-behavior relationships in all three behavioral tasks in the Healthy Brain Network sample and all three behavioral tasks in the Yale Attention sample. The*Nearest Neighbors*model significantly fit the data for the working memory task in the Healthy Brain Network sample and the sustained attention (gradCPT) and working memory (VSTM) tasks in the Yale Attention sample. We next wanted to ask about the relationship between a different type of phenotypic measure—age—and neural synchrony. Do participants more similar in age show higher ISC as predicted by the*Nearest Neighbors*model, or do older (or younger) participants show more similar ISC as predicted by the*AnnaK*model? One might predict similarity in participant age to exhibit a*Nearest Neighbors*-style relationship, particularly in the Healthy Brain Network sample, due to its inclusion of ages that span the course of adolescence (6–22). For instance, a 7-year-old and a 21-year-old are likely to have different interpretations of a narrative stimulus, and these interpretations may be reflected in different fMRI time-courses during movie-watching. Unlike the developmental Healthy Brain Network sample, the Yale Attention dataset is a sample of young adults (aged 18–36). The brain undergoes significant changes during development, and shows relative stability during early adulthood (Bethlehem et al., 2022), and consequently we might anticipate less age-related variability in video processing in the Yale sample, potentially resulting in little age-related differences in neural synchrony. This in turn would result in a poor fit for both the*AnnaK*and*Nearest Neighbors*models.

Examining the results of the age-specific intersubject RSA (Fig. 4), we found significant evidence of the*Nearest Neighbors*model in the Healthy Brain Network sample (251 significant nodes, 250 surviving multiple comparisons correction, familywise*p*< .0001). In line with our hypothesis, this implies that there is greater neural synchrony among participants that are closer in age. As for the*AnnaK*model, we observe, in contrast to our behavioral findings, primarily*negative*correlations between brain similarity and*AnnaK*-defined behavioral similarity (165 significant nodes, 145 surviving multiple comparisons correction, familywise*p*< .0001). This negative correlation implies that the older participants get, the more*dissimilar*they appear from each other, in terms of brain synchrony. In the Yale Attention dataset, neither the*AnnaK*nor the*Nearest Neighbors*models well described the relationship between brain and age similarity (13 and 14 significant nodes respectively, 0 surviving multiple comparisons correction, familywise*p*-values >.47).

In sum, both the*AnnaK*model and the*Nearest Neighbors*model described the relationship between age and intersubject correlation in the Healthy Brain Network sample. This raises the possibility, initially proposed byFinn et al. (2020), that for some phenotypic measures, modeling phenotypic similarity as a combination of the*AnnaK*and*Nearest Neighbors*models might yield the best fit to brain similarity (for instance, by using the formulaabs(i-j)* mean(i,j)). In the Yale Attention dataset, neither model was a good fit, although more research is necessary to determine if this is due to the constrained age range of this sample or some other factor.

#### 3.3.7 Intersubject representational similarity analysis in Healthy Brain Network subsamples

The Healthy Brain Network sample contains a large age range (6–22 years). Our main analysis controlled for age through residualization and did not assess if brain-behavior relationships differed as a function of age. To test this, we split our sample into thirds, based on the 33^rd^and 66^th^percentile ages (9.94, rounded to 10 years and 14.03 rounded to 14 years) to provide a roughly even split of participants by age bin. We then replicated the IS-RSA in participants [6,10) (n = 138), [10,14) (n = 130) and [14,18) (n = 132, same control matrices as prior analysis, seeMethods). In the behavioral tasks and assessments, the results in the different age bins appeared largely consistent with those observed in the full sample (Supplementary Fig. 11). One notable difference across age groups, however, is the measure which shows the most robust*AnnaK*effect. Language and working memory demonstrated the strongest effects at ages 6–10 and 10–14, respectively. This pattern of results is possibly due to differences in variability in the measures across the age bins, with greater variability providing more power to detect effects. For instance, the 6–10 age group demonstrates greater variability in the language measure (TOWRE) compared to the other two groups (**ages 6**–**10**standard deviation: 19.26,**ages 10**–**14**standard deviation: 16.54,**ages 14**–**18**standard deviation: 13.94).

In contrast to the generally consistent brain-behavior relationships, the association between brain similarity and age varies by age group. In participants aged 6–10 years, there is a*positive AnnaK*effect, meaning that neural synchrony increases with age. In the participants aged 10–14, there is a weaker positive AnnaK effect. Finally, in the oldest group, participants aged 14–18, there is a negative*AnnaK*effect, meaning that neural synchrony decreases with age.

In addition to the wide age range included in the Healthy Brain Network dataset, there are also a diverse range of clinical diagnoses represented in this sample. Do brain-behavior relationships vary as a function of diagnosis? Because the current sample was not large enough to perform a comprehensive investigation of each diagnosis, we chose to replicate the IS-RSA analyses with a subsample of participants diagnosed with ADHD, the most common clinical diagnosis in the sample (n = 55;Supplementary Fig. 12). We find similar results to that of the full sample, with evidence of an*AnnaK*effect, where high scorers appear more similar to high scorers, with the strongest effect in the working memory measure (familywise*p*< .0001). These results suggest that characterization of brain-behavior relationships with the AnnaK model is robust to ADHD diagnosis.

## 4 Discussion

Recent work hypothesizes that similarities in behavior are reflected in similar BOLD responses to movies, but that this relationship does not always hold constant across the behavioral spectrum (Finn et al., 2020). Specifically, Finn and colleagues found that participants who scored highly on a test of working memory showed greater neural synchrony with other high scorers, while individuals who scored low appear less synchronized across the board. In this instance, neural similarity is indicative of behavioral similarity, but only in high-scoring participants. How does the relationship between neural similarity and behavior play out in other behavioral domains?

In the present study, we investigate this question, examining brain-behavior relationships in eight measures across samples from two datasets: the Healthy Brain Network dataset (measures reflecting working memory, attention/executive function, language, depression/anxiety, and social function) and the Yale Attention dataset (behavioral tasks scores, measuring working memory, sustained attention, and divided attention/tracking, respectively). Using intersubject-RSA, we tested two models of neural synchrony: 1. the*Nearest Neighbors*model, stating that individuals will look similar to those nearby on some behavioral scale and 2. the*AnnaK*model, stating that high scorers will look like other high scorers while low scorers will show greater variability (or vice versa). Overall, our analyses revealed greater evidence for the*AnnaK*model of brain-behavior relationships, particularly for the working memory task in the Healthy Brain Network Dataset and the sustained attention task in the Yale Attention dataset. Put another way, results here suggest that in tasks across datasets and behavioral measures, high scorers tend to resemble high scorers, whereas low scorers appear dissimilar from everyone else. While the*Nearest Neighbors*model also fits the data above chance for four of the eight measures tested, in no behavioral measure did it clearly outperform the*AnnaK*model when examining number of significant nodes and family-wise*p*-values. This finding is striking considering that the*Nearest Neighbors*model is arguably a more common way of operationalizing the association between brain activity and cognitive performance. Our results replicate previous observations of*AnnaK*-style ISC-behavior associations (Finn et al., 2020) and underscore the importance of considering the meta-relationship between brain and behavior. By explicitly testing how brain and behavior relate, researchers can avoid the potential pitfall of erroneously reporting an absence of a relationship due to implicitly assuming a certain type of relationship in their analyses.

An advantage of analyzing data from two datasets is that we can assess the replicability of*AnnaK*effects across participant populations (children and adolescents vs. young adults), naturalistic stimuli (a narrative*Despicable Me*movie clip vs. the non-narrative*Inscapes*film), and phenotypic measures (e.g., a list-sorting verbal working memory task vs. visual short-term memory task). We found evidence for*AnnaK*-style relationships in both datasets, suggesting that such effects are not confined to adult populations, uniquely evoked by narrative movies, or specific to one set of phenotypic measures. It is more difficult to interpret differences across datasets than consistency between them. For example, although we see stronger evidence for the*AnnaK*model in the Healthy Brain Network sample than the Yale Attention sample, it is unclear what drives this difference. It could arise from the age range and/or heterogeneity of the participant sample (individuals recruited due to perceived clinical concerns vs. a non-clinical sample), the choice of movie, the study design (one vs. two scan sessions; seeSupplementary Fig. 13for analyses by session), the scan site (multiple sites in HBN vs. one site in Yale Attention) or imaging parameters, and/or noise. Future work characterizing effects of age, naturalistic stimulus, phenotypic measure, and data type and amount will help address these questions.

When relating intersubject-RSA to age, we observed evidence for the*Nearest Neighbors*and*AnnaK*models in the Healthy Brain Network sample only. These results suggest that, in the Healthy Brain Network dataset, younger participants and participants closer in age show greater neural synchrony. While this result may seem curious when juxtaposed with our behavioral findings, it is in line with prior work demonstrating that ISC as measured with EEG decreases with age (participants aged 5–44) (Petroni et al., 2018) and ISC as measured with fMRI decreases with age (participants aged 18–88) (Campbell et al., 2015). However, existing literature is somewhat mixed with regard to the association between neural synchrony and age, with some evidence suggesting that adults exhibit greater ISC compared to children (Cantlon & Li, 2013), particularly in the default mode network (Moraczewski et al., 2020). Another study analyzing data from the Healthy Brain Network found, in line with our findings here, more areas of the brain where ISC was greater in younger participants than older participants (Cohen et al., 2022). Inconsistent with the current findings, however, the same study reports higher ISC among older participants in the auditory cortex (Cohen et al., 2022) and another reports greater synchrony among older participants across the cortex more generally (Camacho et al., 2023). One factor that may contribute to this discrepancy is our use of a relatively conservative motion threshold (mean framewise displacement <.15 mm) in determining our sample. While this criterion should help safeguard against spurious motion-driven effects, we cannot rule out the possibility that motion in this sample is related to another variable affecting ISC (Power et al., 2012). Additionally, our analyses include all ages available in the Healthy Brain Network dataset (ages 6–22 years); when excluding participants 16 years and older (as inCamacho et al., 2023), we observe a primarily positive relationship between age and intersubject correlation (seeSupplementary Fig. 14). Consequently, future work remains to elucidate how ISC changes across development.

The*AnnaK*model may help us to characterize the nature of brain-behavior similarity, but future research is needed to determine the source of increased variance for one side of a behavioral scale. How can we understand features characteristic of the end of the spectrum marked by greater heterogeneity? One approach which may prove useful is the classification of behavioral performance. Among individuals with the same summary score on a behavioral task, there may be clusters of participants that can be differentiated based on their patterns of responses. For instance, perhaps some individuals take longer to recover after making errors. A separate group of participants may quickly regain focus after errors but show a marked deterioration in performance near the end of the task, as their concentration wanes. If neural similarity is higher within these clusters, but lower across them, this could help explain why there is more variation in brain activity among low-scoring individuals. In this hypothetical case, the original behavioral metric does not align well with neural variability and consequently obscures a*Nearest-Neighbors*relationship. However, there may be a limit to how well we can characterize this variability using individual differences approaches, if low-scoring individuals appear not only dissimilar to each other, but dissimilar to themselves. This may prove to be the case, considering recent work showing that intra-individual variability is associated with worse task performance, both in terms of session-to-session whole-brain functional connectivity (Corriveau et al., 2022) as well as trial-by-trial patterns of hippocampal activity (Poh et al., 2022). In other words, in some instances, there may be a “true”*AnnaK*effect, rather than a*Nearest Neighbors*effect, which is obfuscated by an inadequate behavioral scale.

Making use of two open datasets, we demonstrated an*AnnaK*effect in disparate samples in measures of attention and cognition. Moving forward, future work can leverage open data to further test hypotheses regarding the meta-relationship between brain and behavior in diverse samples that researchers might not have access to at their individual institutions. Collection of continuous measures is key to this effort, as it enables IS-RSA and other data-driven approaches that rely on testing individual differences on a spectrum rather than simple contrasts comparing one group to another (i.e., testing for differences in patient vs. controls). Furthermore, future research might investigate similarity of other aspects of brain function and organization, such as correlation of functional connectivity patterns or agreement between functional subnetworks (Glerean et al., 2016).

The present study aimed to further elucidate the connection between similarity in brain activity and similarity in behavior. Using intersubject representational similarity analysis, we conceptually replicated prior work and empirically assessed assumptions in cognitive neuroscience research which may otherwise go untested. In continuing to map the space of neural and behavioral similarity, future work will reveal the structure governing how neural activity produces our idiosyncrasies as individuals.

## Supplementary Material

Supplementary Material

## Data Availability

Healthy Brain Network data are available athttp://fcon_1000.projects.nitrc.org/indi/cmi_healthy_brain_network/. Yale Attention data are available athttps://nda.nih.gov/edit_collection.html?id=2402. Analysis code is available athttps://github.com/tchamberlain/nn_annak.
