# Supplementary material for: High performers demonstrate greater neural synchrony than low performers across behavioral domains
Source: Imaging Neurosci (Camb). 2024 Apr 15;2:imag-2-00128. doi: 10.1162/imag_a_00128 (PMC12247615; doi:10.1162/imag_a_00128)
Supplement: Supplementary Material [file imag_a_00128-supp.pdf]

Supplementary Material

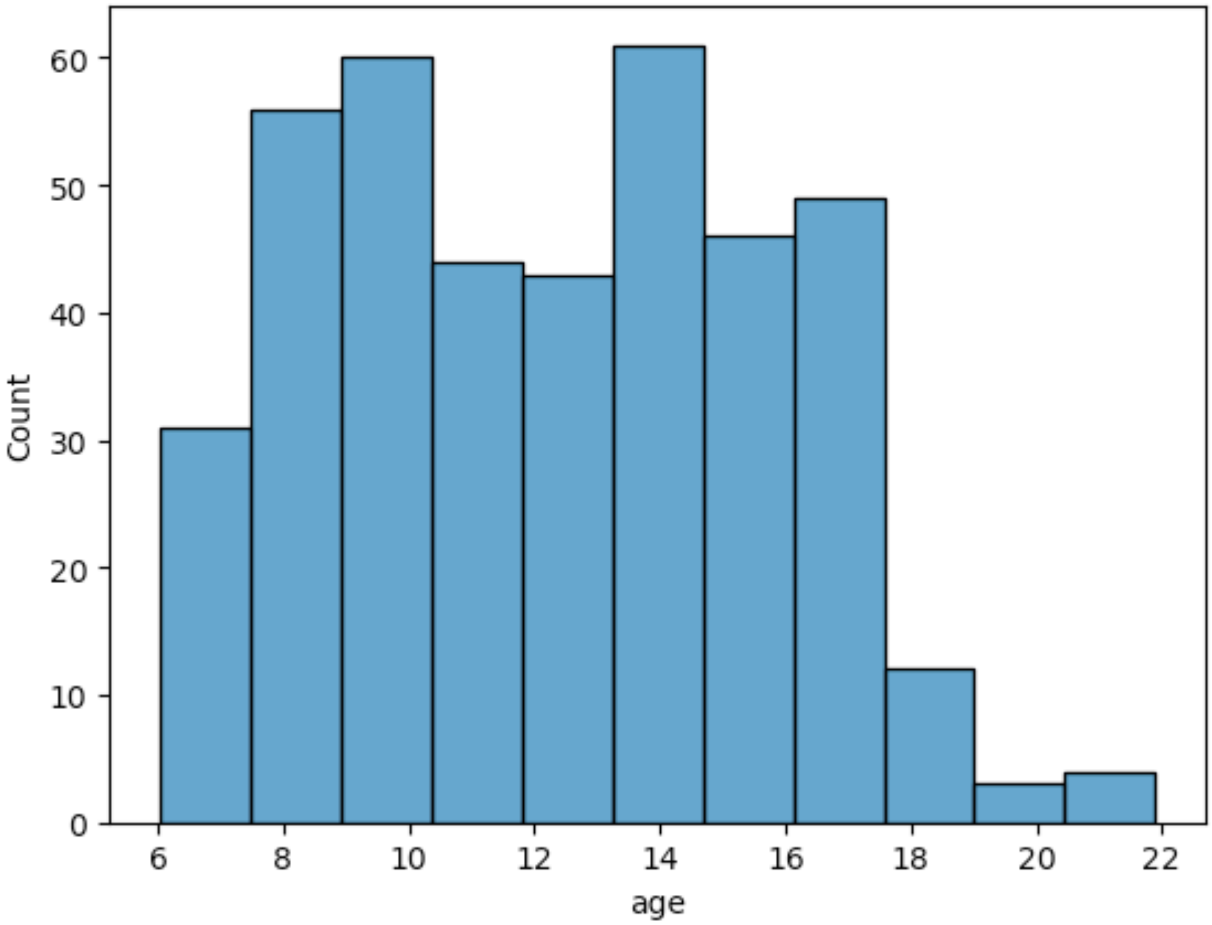

Supplementary Figure 1: Histogram of age of included participants in the Healthy Brain Network sample (n=409).

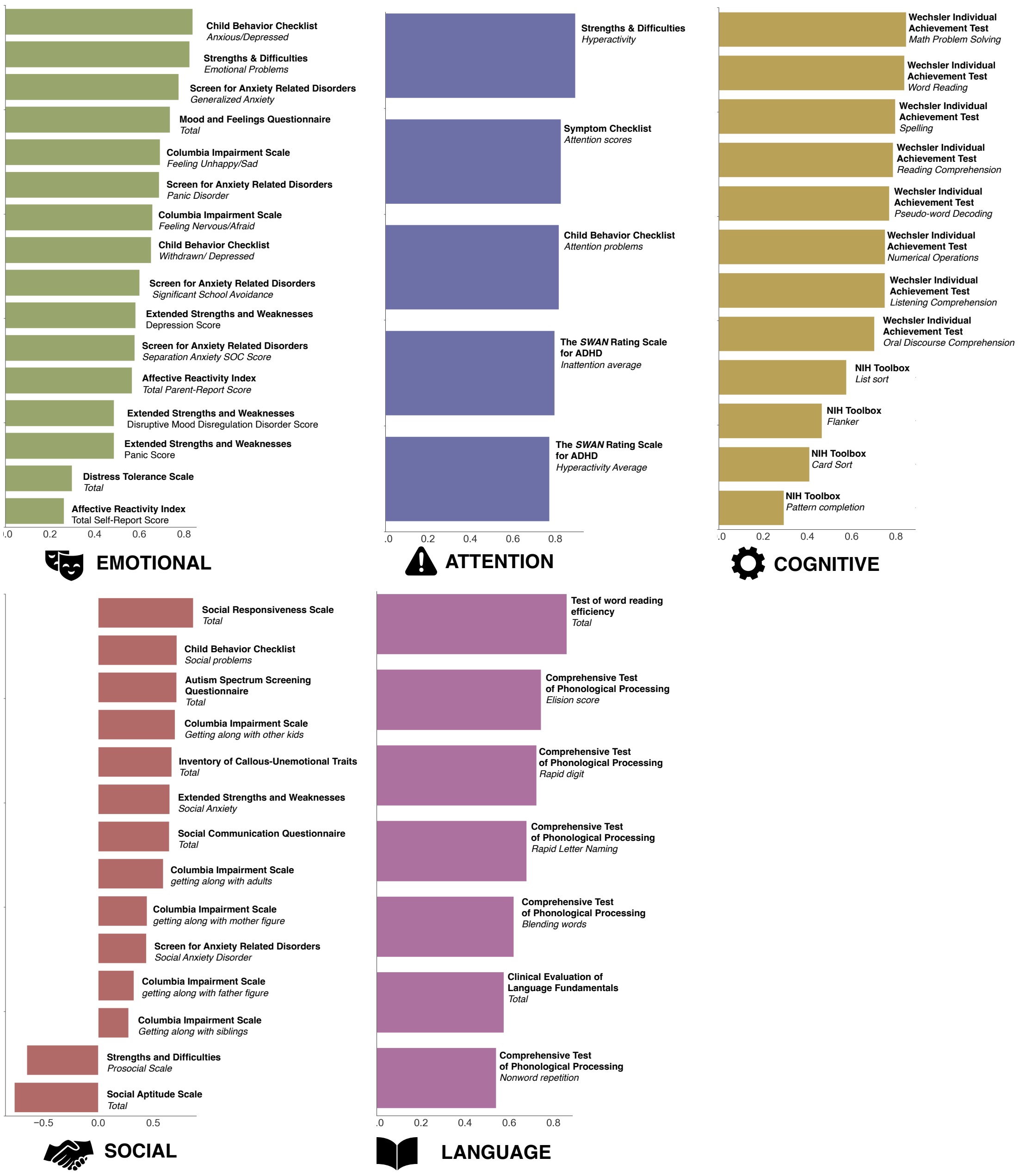

Supplementary Figure 2: First principal component of five phenotypic domains in the Healthy Brain Network sample. The contribution of each variable to the first principal component of each of the five domains of behavior analyzed in the Healthy Brain Network sample. In each plot, the x-axis shows the correlation between the original measure and the first principal component. In Supplementary Figure 5, Emotion, Attention, and Social scores are reverse coded for visualization, such that a greater value indicates better function in each respective domain

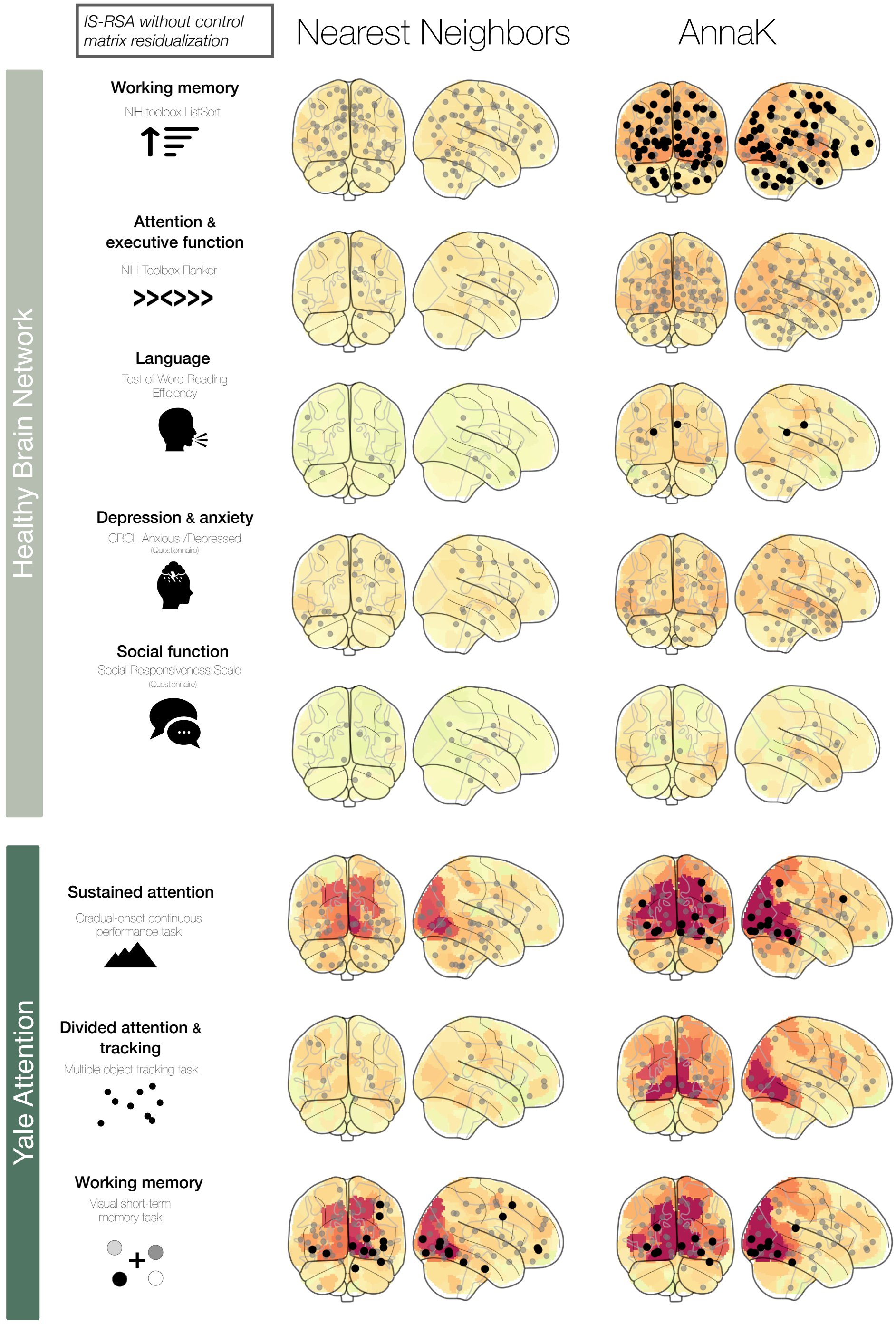

Supplementary Figure 3: Intersubject RSA (without control residualization). IS-RSA results for each measure. Unlike in the analysis in the main text (Figure 2), the analysis shown here does not control for age, sex, or motion. Plotting conventions are the same as figures in the main text (see Figure 2).

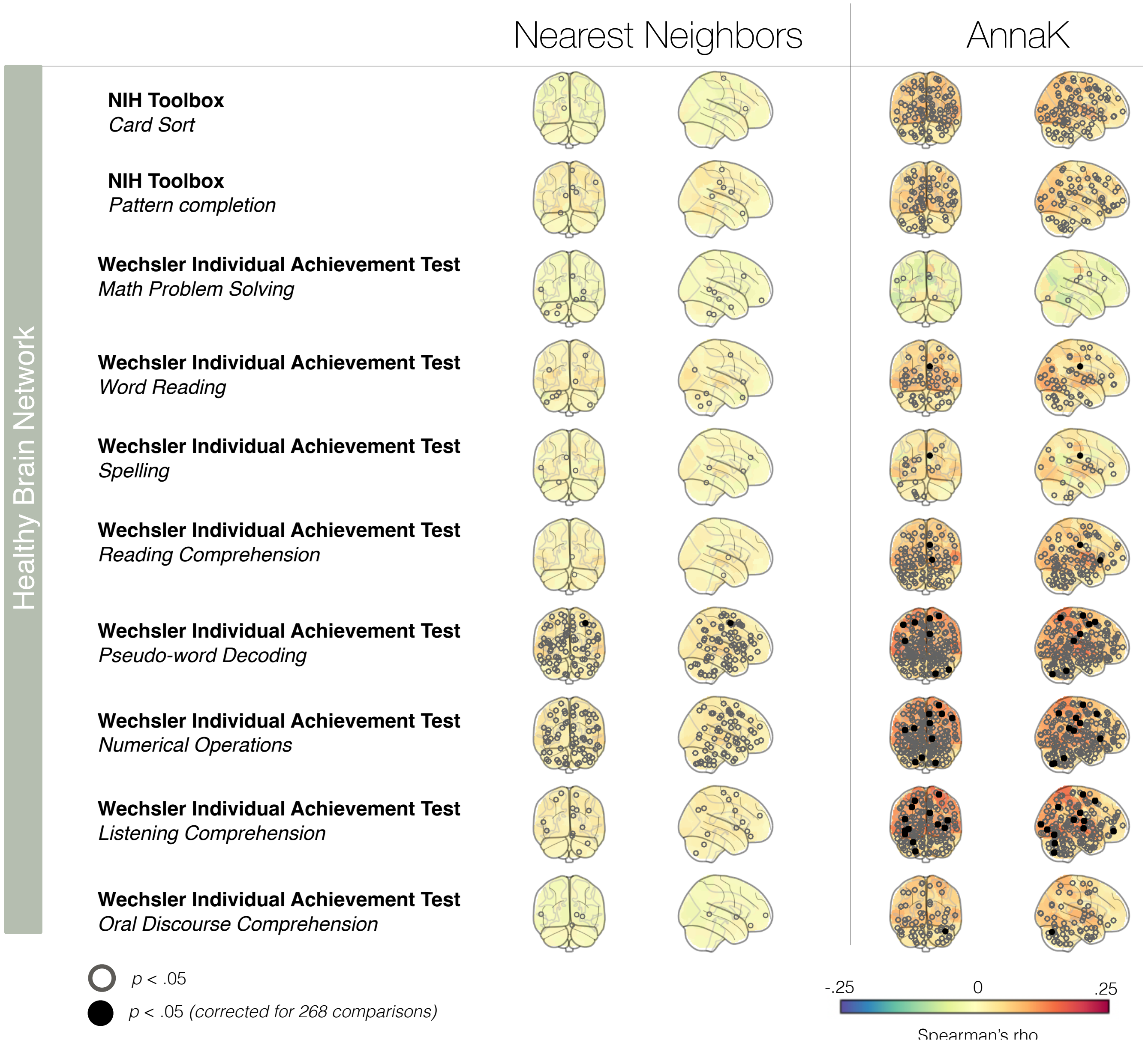

Supplementary Figure 4: Intersubject RSA (Healthy Brain Network cognitive measures). IS-RSA results for each variable in the "cognitive domain" PC. Plotting conventions are the same as figures in the main text (see Figure 2).

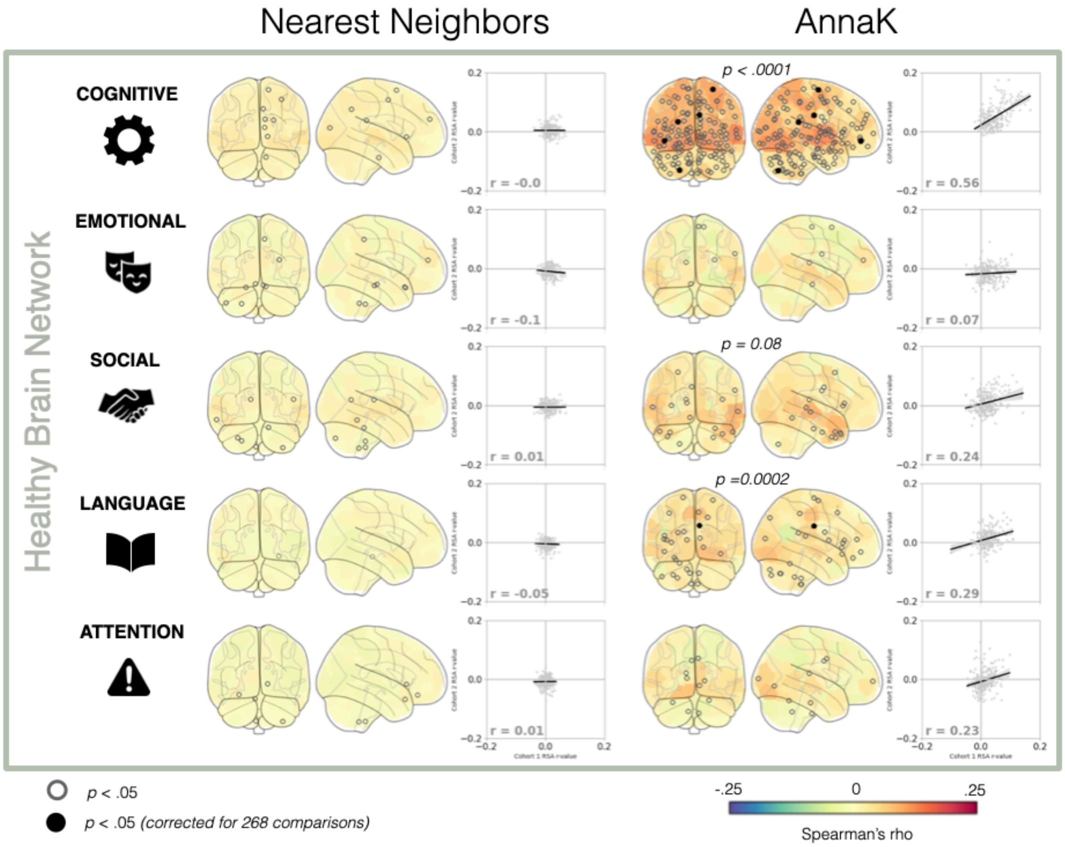

Supplementary Figure 5: Intersubject RSA with first principal components. IS-RSA results for age in the Healthy Brain Network sample, using the first principal components of the PCA measures derived for each behavioral domain (for loadings of the first principal component see Supplementary Figure 2).

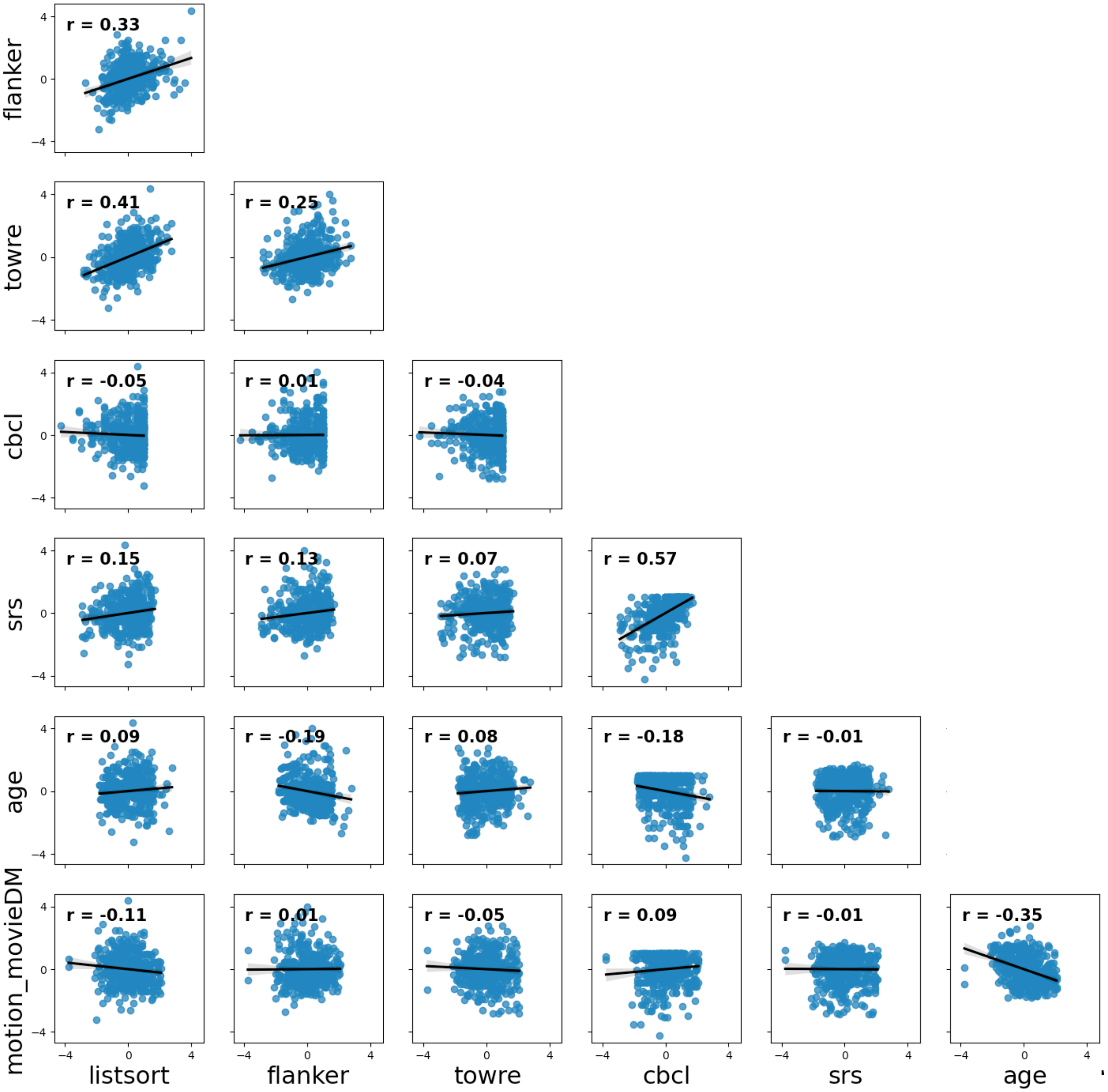

Supplementary Figure 6: Associations between Healthy Brain Network measures.
Scatterplots showing the relationship between z-scored measures in the Healthy Brain Network biobank. The r-value is the Pearson correlation between the two measures.

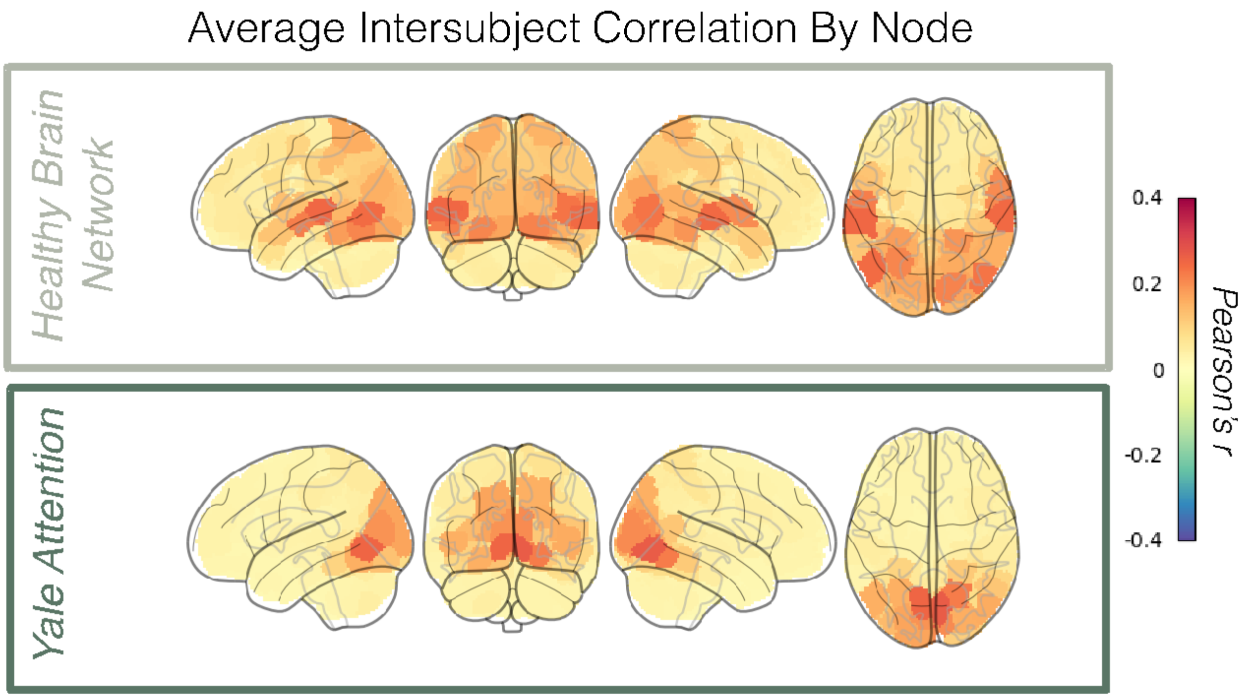

Supplementary Figure 7: Intersubject correlation. Here we show pairwise intersubject correlation, calculated separately in each node in our 268-node parcellation, and averaged across all participants. Intersubject correlation is defined as the Pearson correlation between the BOLD time series of two different participants for a given node.

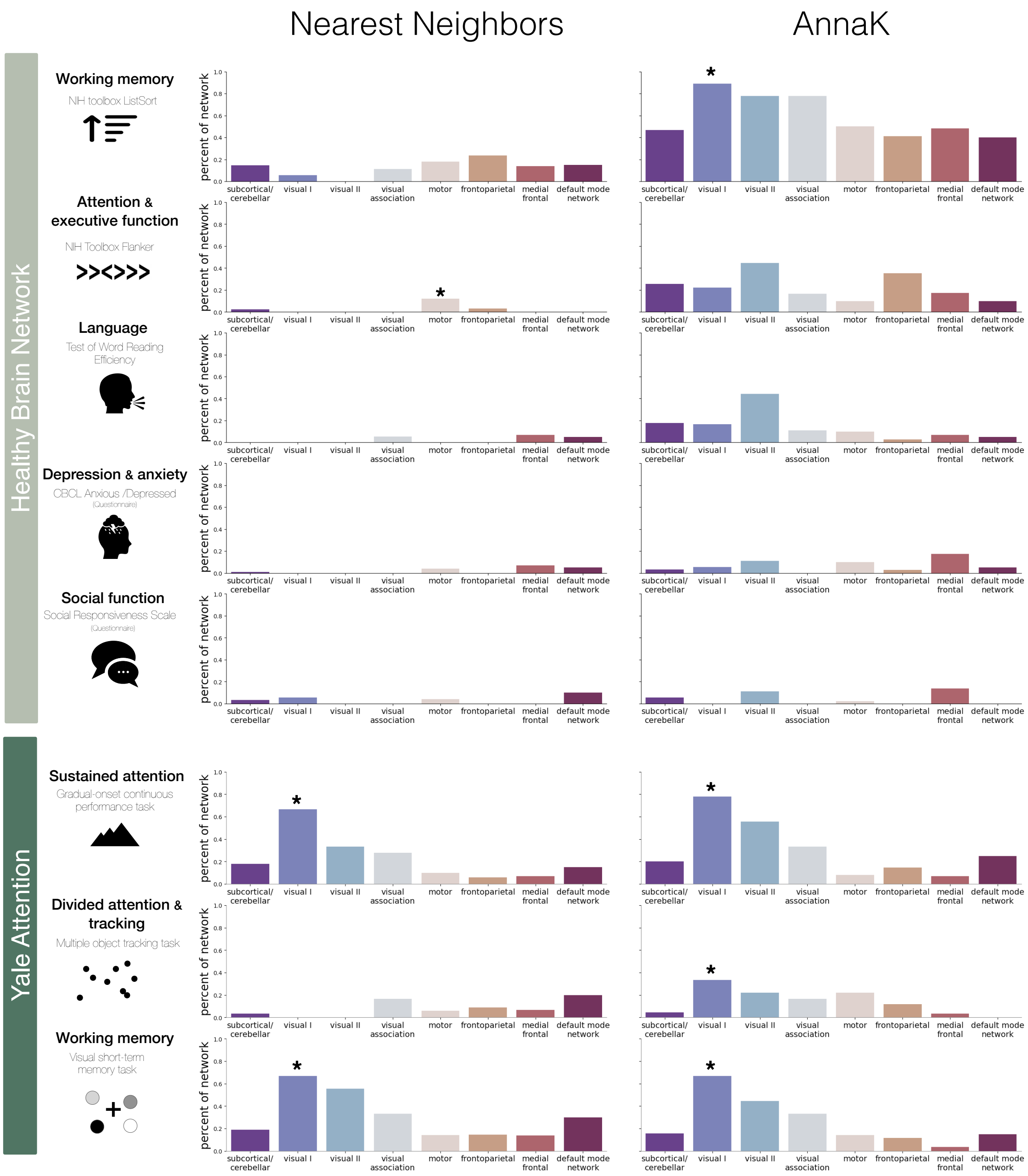

Supplementary Figure 8: Intersubject RSA results by network. For each behavioral task, for each model, we show the percent of nodes in a given network that show significant representational similarity. Stars indicate that the given network demonstrates a greater number of nodes than expected by chance, as determined by permutation testing (p<.05 corrected for 8 comparisons).

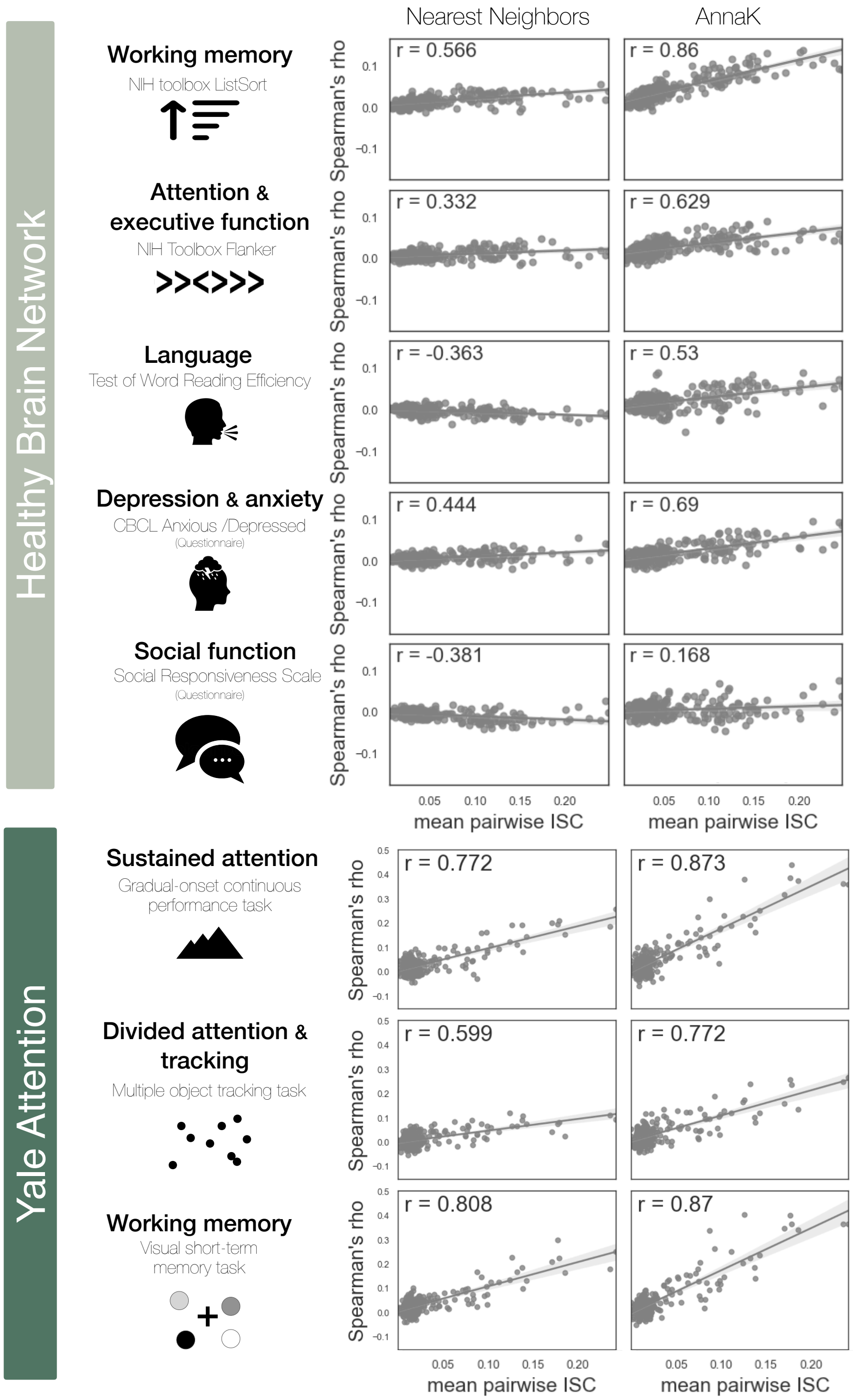

Supplementary Figure 9: Correlation between IS-RSA Spearman Rho and ISC by node. Each point on each scatterplot is one of 268 nodes in the whole-brain parcellation. The y-axis shows the rho-value representing model fit in that node (brain similarity correlated with behavioral similarity for a given model). The x-axis shows the mean pairwise intersubject correlation in the node.

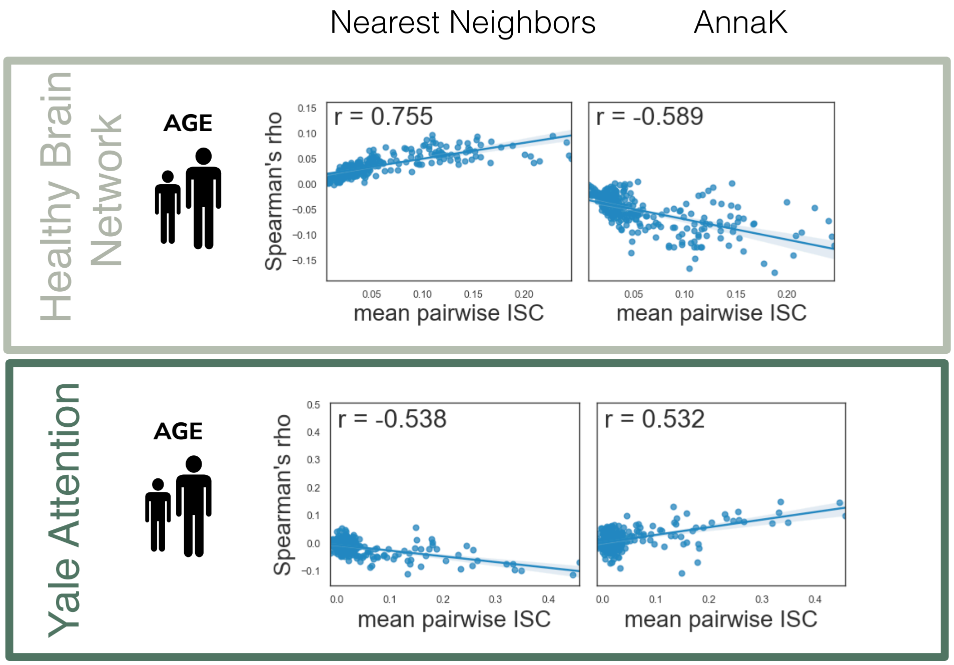

Supplementary Figure 10: Correlation between IS-RSA Spearman Rho and ISC by node. Each point on each scatterplot is one of 268 nodes in the whole-brain parcellation. The y-axis shows the *rho*-value representing model fit in that node (brain similarity correlated with behavioral similarity for a given model). The x-axis shows the mean pairwise intersubject correlation in the node.

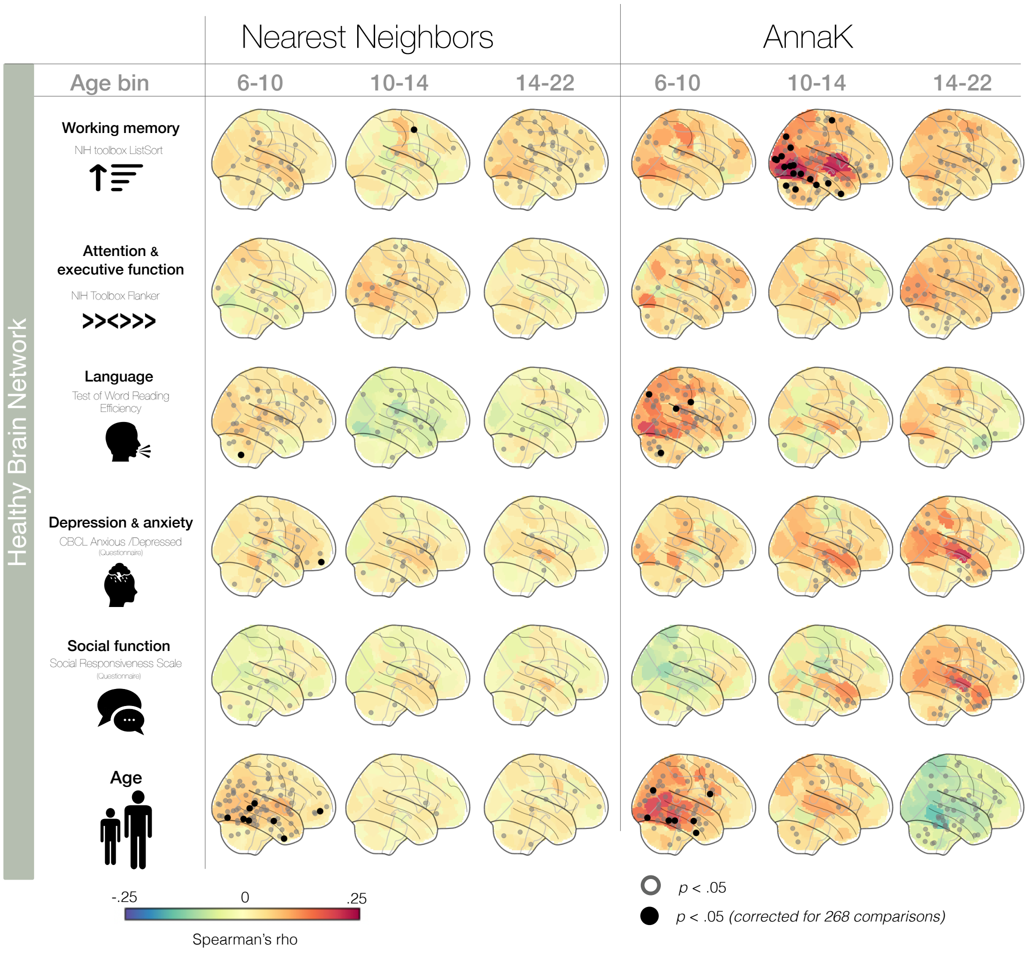

Supplementary Figure 11: Intersubject RSA by age. IS-RSA results three sub-samples of the HBN sample: participants ages 6-10, participants ages 10-14, and participants ages 14-18. Plotting conventions are the same as figures in the main text (see Figure 2).

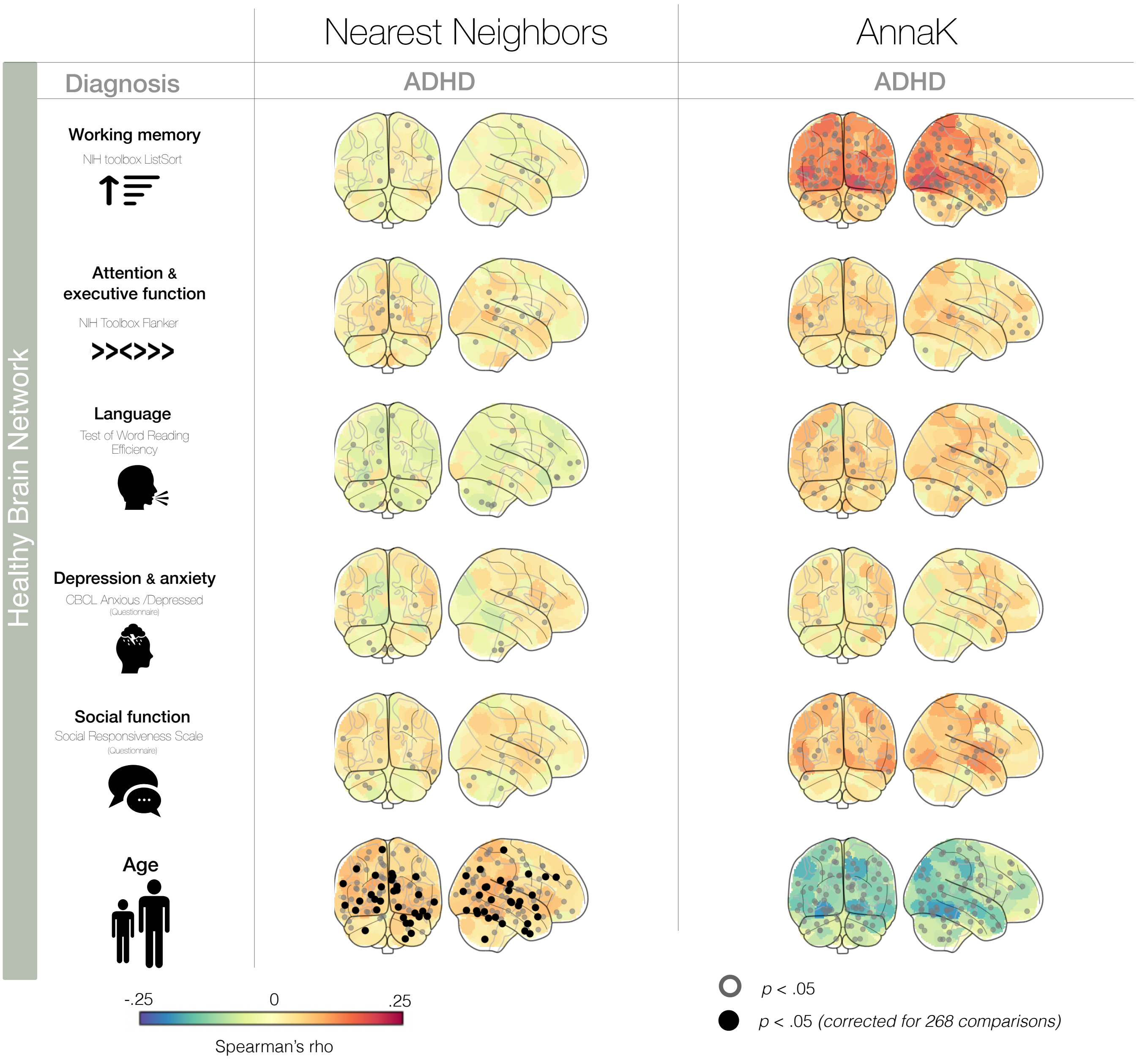

Supplementary Figure 12: Intersubject RSA in participants with an ADHD diagnosis. IS-RSA results a sub-sample of the HBN sample: participants with an ADHD diagnosis (i.e., diagnosed with one of the following, and no other diagnosis: ADHD-Inattentive Type, ADHD-Combined Type, Other Specified Attention-Deficit/Hyperactivity Disorder, ADHD-Hyperactive/Impulsive Type). Plotting conventions are the same as figures in the main text (see Figure 2).

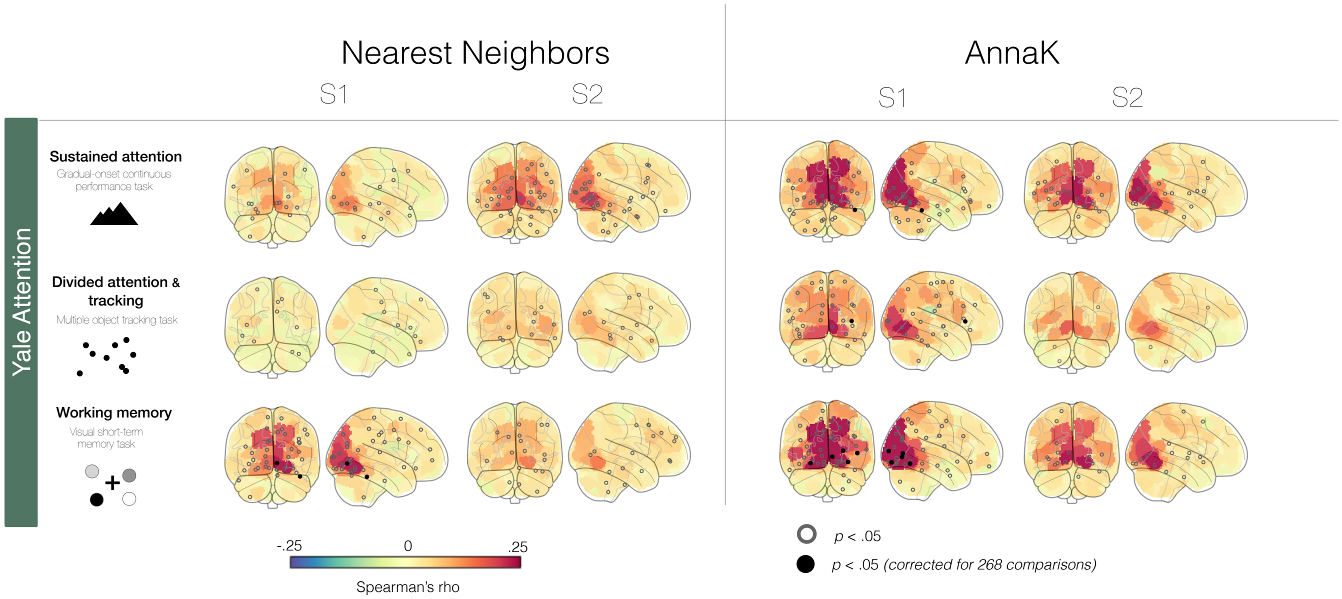

Supplementary Figure 13: Intersubject RSA (Yale Attention). IS-RSA results for each variable in Yale Attention dataset, separated by movie session (S1 is the first viewing of the film, and S2 is the second). Plotting conventions are the same as figures in the main text (see Figure 2).

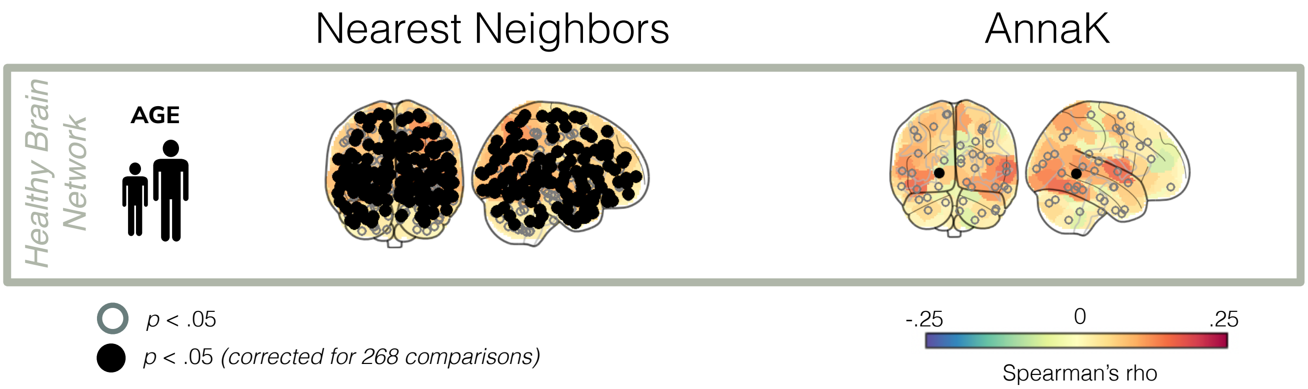

Supplementary Figure 14: Age Intersubject RSA excluding ages 16 and above. IS-RSA results for age in the Healthy Brain Network sample, but excluding participants aged 16 years and older (the main text includes all available ages, 6-22). Plotting conventions are the same as figures in the main text (see Figure 2).
